# Chicken Interferon-induced Protein with Tetratricopeptide Repeats 5 Antagonizes Replication of RNA Viruses

**DOI:** 10.1038/s41598-018-24905-y

**Published:** 2018-05-01

**Authors:** Diwakar Santhakumar, Mohammed Abdel Mohsen Shahaat Rohaim, Hussein A. Hussein, Pippa Hawes, Helena Lage Ferreira, Shahriar Behboudi, Munir Iqbal, Venugopal Nair, Clarice W. Arns, Muhammad Munir

**Affiliations:** 1 Division of Biomedical and Life Sciences, Faculty of Health and Medicine, Lancaster University, Lancaster, LA1 4YG UK; 2 The Pirbright Institute, Woking, Surrey, GU24 0NF UK; 3 Department of Virology, Faculty of Veterinary Medicine, Cairo University, Giza, 12211 Egypt; 4 Universidade de São Paulo, Campus de Pirassununga, São Paulo, Brazil; 5 Department of Pathology and Infectious Disease, School of Veterinary Medicine, University of Surrey, Surrey, UK; 6 Institute of Biology, University of Campinas, São Paulo, Brazil

## Abstract

The intracellular actions of interferon (IFN)-regulated proteins, including IFN-induced proteins with tetratricopeptide repeats (IFITs), attribute a major component of the protective antiviral host defense. Here we applied genomics approaches to annotate the chicken IFIT locus and currently identified a single IFIT (chIFIT5) gene. The profound transcriptional level of this effector of innate immunity was mapped within its unique *cis-acting* elements. This highly virus- and IFN-responsive chIFIT5 protein interacted with negative sense viral RNA structures that carried a triphosphate group on its 5′ terminus (ppp-RNA). This interaction reduced the replication of RNA viruses in lentivirus-mediated IFIT5-stable chicken fibroblasts whereas CRISPR/Cas9-edited chIFIT5 gene knockout fibroblasts supported the replication of RNA viruses. Finally, we generated mosaic transgenic chicken embryos stably expressing chIFIT5 protein or knocked-down for endogenous chIFIT5 gene. Replication kinetics of RNA viruses in these transgenic chicken embryos demonstrated the antiviral potential of chIFIT5 *in ovo*. Taken together, these findings propose that IFIT5 specifically antagonize RNA viruses by sequestering viral nucleic acids in chickens, which are unique in innate immune sensing and responses to viruses of both poultry and human health significance.

## Introduction

Innate immune responses, primarily triggered by interferons (IFNs) and their antiviral effectors, can establish an extremely potent antiviral state to efficiently restrict virus replication and virus-induced pathologies in the susceptible host^1^. To initiate these innate responses, viruses are detected by the host pathogen recognition receptors (PRRs) through recognition of viral molecular signatures known as pathogen associated molecular patterns (PAMPs)^1^. PAMPs associated with most viruses include viral double-stranded RNA (dsRNA), which is produced as replicative intermediate and single-stranded adenosine/uridine (AU)-rich regions^2^. These PAMPs effectively activate cascades of signalling events that culminate in the production of IFNs in virus-infected cells. The released IFNs transcriptionally activate hundreds of IFN-stimulated genes (ISGs) in uninfected neighbouring cells that instigate direct or indirect antiviral activities^3,4^. Well-studied examples of these antiviral effectors are dsRNA-activated protein kinase R and 2′-5′ oligoadenylate synthetase which bind to dsRNA, and interferon induced proteins with tetratricopeptide repeats (IFIT)-1 and -5 that bind to 5′-triphosphate containing RNA^1,5^.

IFIT genes are evolutionary conserved and are originated possibly by gene duplication^6^. All members of the IFIT family consist of multiple tetratricopeptide repeats (TPR) throughout the length of the protein that are mainly responsible for the protein-protein interaction and assembly of larger protein complexes^7,8^. Most mammals encode several IFIT genes including IFIT1/ISG56, IFIT2/ISG54, IFIT3/ISG60 and IFIT5/ISG58, however, mice and rats lack IFIT5 and horses lack IFIT1^6^. Amongst all known members of the family, IFIT1 and IFIT5 are highly responsive both at the transcription and translation levels to diverse cellular stresses including those induced by dsRNA, virus infections and lipopolysaccharides^1,5^. The IFIT proteins family is responsible for diverse array of cellular activities including nucleic acid sensing and direct antiviral effects^9^. Specifically, IFIT1 is proposed to be a negative-feedback regulator of virus-triggered induction of type I IFNs and cellular antiviral responses^10^ whereas IFIT5 potentiate antiviral signalling^11^. Additionally, mammalian IFIT5 can sense and bind to numerous short cellular RNAs such as initiator tRNA, and these interactions are mapped across the protein surface^12^.

The most prominent features of IFIT5 proteins are attributed to their involvements in the inhibition of virus replication through nucleic acid sensing and leading to possible inhibition of translation^8,9^. IFIT5 protein, in addition to IFIT1, discriminates the cellular and viral mRNA for initiating downstream antiviral activities by recognition of discrete features at the 5′ termini^7,13^. Most eukaryotic cellular ribosomal and transfer RNAs (rRNAs and tRNAs) carry monophosphate at 5′-termini whereas messenger RNAs (mRNAs) bear N7-methlguanosine cap (cap0) attached to the first base through 5′-5′ triphosphate bridge that recruits cellular factors involved in RNA processing and translation initiation^14,15^. Additionally, in most higher eukaryotes methylation occurs at the 2′-O position of the first or second base yielding the cap1 (m^7^GpppNmN) or cap2 (m^7^GpppNmNm) structures, respectively^14^. Although cap1 and cap2 are not crucial for mRNA translation, human and murine IFIT1 protein can inhibit translation of cap1-lacking mRNA^16,17^. These features of cellular RNAs are also mimicked by several viruses as countermeasure strategies^17^. However, viral genomic and subgenomic RNA of negative sense single-stranded viruses such as influenza A viruses (IAV) and Newcastle disease viruses (NDV) bear triphosphate at the 5′-termini^14^. These PAMPs are sensed by cellular PRRs and initiate innate immune responses, which ultimately restrict virus growth^1,5^.

Significant genetic, functional and structural features have been recently attributed to the mammalian IFIT genes and proteins^16–19^. There is limited information available on the repertoires of cellular proteins that recognizes different populations of viral nucleic acid in avian species especially in chickens which differ significantly in mounting innate immune responses and are infected by pathogens that continuously pose zoonotic threats to public health^5^. Here, we have genetically characterized chicken IFIT and revealed that chickens, in contrast to other vertebrates, encode only one IFIT gene (chIFIT5). The chIFIT5 gene was transcriptionally highly responsive to both type I IFNs and RNA viruses (IAV and NDV) and interestingly this responsiveness was mapped to ISRE motif in the *cis-acting* elements. We also demonstrated that chIFIT5 specifically interacts with ssRNA carrying 5′-ppp moiety. Through this interaction, chIFIT5 applies strong antiviral activities in lentivirus-transduced stable cell lines whereas CRISPR/Cas9-mediated chIFIT5 knockout promoted virus replication. Finally, employing the RCAS-based retroviral gene transfer vector system^20^, we generated transgenic chicken embryos expressing chIFIT5 and demonstrated its antiviral potential *in ovo*. These findings were further evaluated by RCAS-mediated gene silencing in developing transgenic chicken embryos by assessing the replication kinetics of RNA viruses. These analyses provide evidence of the presence of a functional homologue of IFIT5 and expand our understanding on the breaths and dynamics of nucleic acid sensing in chicken.

## Results

### Genomic annotation of IFIT locus revealed that chicken genome encodes IFIT5 gene

**Figure 1:**
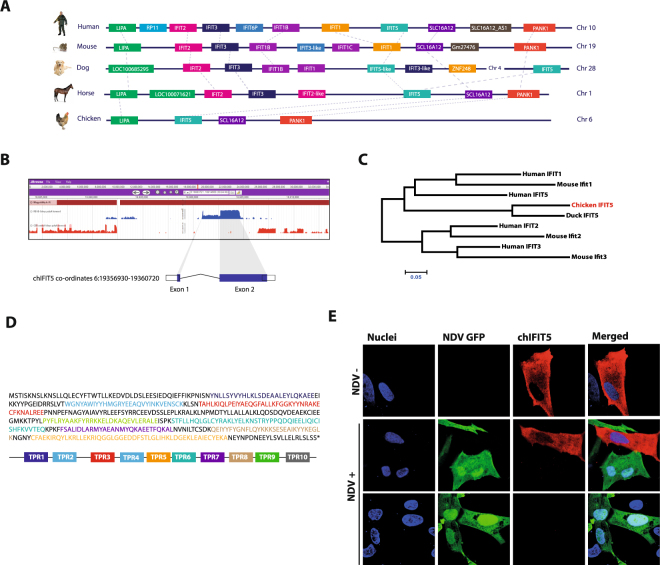
Genomic architecture along with relative loci around IFIT genes in human, mouse, dog, horse and chicken, and gene annotation in chicken. (**A**) The IFIT locus in compared species is flanked upstream with LIPA gene and downstream with PANK1 gene. Direct syntenic analysis identified a single IFIT5 gene in chicken compared to four in other species aslong with other pseudogenes. (**B**) Transcriptomics profiling of chicken primary fibroblasts which were mock-infected or infected with RB1B strain of MDV. Blue bar represents the transcript in the current chicken genome assembly whereas the red bar represents the mappability of the transcript to the chicken genome. A final transcript from MDV-infected data and gene characterization is shown at the bottom. (**C**) Phylogenetic analysis of IFIT genes in different species. Based on the clustering patterns and sequence homologies, the single identified gene clustered closer to IFIT5 of duck and human. (**D**) Putative tetratricopeptide repeats (TPR) showing characteristic features of IFIT proteins. (**E**) Expression and subcellular distribution of chIFIT5 in chicken embryo fibroblasts. Chicken cells were transfected with 500 ng of mammalian expression vectors encoding V5-tagged chIFIT5 for 24 hours and were left untreated (NDV-) or were treated with 1 MOI of NDV-GFP (NDV+) for another 24 hours before fixation, staining for nucleus (blue), chIFIT5 (red) and GFP marker (NDV).

The genome of all major mammalian species (including human, mouse, dog and horse) encodes for IFIT genes, however, these genes are only genetically and functionally characterized in a limited number of species^9,19^. The human genome encodes four IFIT genes (IFIT1, IFIT2, IFIT3 and IFIT5) whereas rats and mice lack IFIT5 and horses lack IFIT1 (Fig. 1A). In addition, several pseudogenes have been identified in different animal species including IFIT1B (in human, mice and rabbits), IFIT1C (in mice), IFIT3-like (in dogs and mice) and IFIT5-like gene (in dogs and rabbits). To identify corresponding IFIT homologues in chicken, initially several IFIT genes from human and mouse were used in the BLAST algorithm in the Ensembl database. Based on high genetic similarity with huIFIT5, only a single gene (*ENSGALT00000010311*) was identified in the chicken genome (Fig. 1A).

The IFIT locus, encoding all IFIT genes, is primarily mapped in between the lysosomal acid lipase/cholesteryl ester hydrolase (LIPA) and pantothenate kinase 1 (PANK1) genes. Since only a single IFIT gene (IFIT5) was identified in the chicken genome, we next examined an approximately 9.0 kb genomic sequence spanning AADN04000218.1 contigs in the chicken chromosome 6 (Chromosome 6: 19,342,835–19,429,041). Immediately adjacent to the LIPA gene, a sequence gap was identified whose estimated length was 400 bps in the Ensembl chicken genome build (Ensembl release 85 - July 2016) (Supplementary Fig. 1A). Long primers (Supplementary Table 1) flanking each end of the gap were used to amplify genomic fragments and subsequent sequencing of the products was used to cover the genomic gap. We used the sequenced fragments as input in WebAUGUSTUS and predicted any possible genes in the entire IFIT locus. Only one gene (IFIT5) was predicted in any strand of the input DNA using chicken as species parameters (Supplementary Fig. 1A). The genomic gap has now been filled with the latest Ensembl chicken genome (Ensembl release 87 - Dec 2016) and no gene other than IFIT5 has been identified (until 17 April 2018).

To confirm the sequence of the cDNA and to identify the genomic structure of the identified IFIT gene, the transcript was amplified from RNA extracted from NDV-infected CEFs. Complete sequence analysis of the gene revealed an open reading frame (ORF) of 1440 bps (479 amino acids excluding stop codon) with high sequence identity (48% and 67%) with the human and duck IFIT5 proteins, respectively (Supplementary Fig. 1B). The identified gene showed a characteristic exon/intron organization where two exons were separated by a few kilobases (kb) long intron (Fig. 1B). The first exon encodes barely a Kozak sequence (CATG) and 5′ untranslated region (UTR). The second exon codes the rest of the ORF (1435 bps) and 3′ untranslated regions (UTR) for chicken IFIT5 gene. Based on the transcriptomic data from Marek’s disease virus (MDV, strain RB1B)-infected CEFs (Fig. 1B) and cDNA sequencing data, a complete IFIT5 gene was annotated. Phylogenetic analysis of characterized chicken (ch) IFIT5 gene with all known human, mouse and duck IFIT genes clustered the chicken gene with duck and human IFIT5 (Fig. 1C). One of the structural hallmarks of all IFIT proteins is the presence of multiple TRP motifs dispersed throughout the length of the protein^6,21^. The consensus chicken, duck and human IFIT5 sequences were used to predict TRPs using NCBI’s Conserved Domain Database. Both duck and human IFIT5 proteins structurally carried ten TRP motifs and two multi-domains whereas chicken IFIT5 encoded eight predicted and ten structure-based TPRs (Fig. 1D).

Taken together, the gene synteny, genetic similarity, genomic architecture and annotation indicate that chickens encode only one IFIT gene compared to at least four in mammals. Based on genetic clustering, it is highly similar to IFIT5 genes of other avian (chickens and ducks) and mammalian species. Moreover, no ortholog for IFIT1, IFIT2, IFIT3 and no pseudogenes were identified in the current Ensembl chicken genome build (Ensembl release 87 - Dec 2016).

### Subcellular distribution of chicken IFIT5 protein

Mouse IFIT1 protein is predominantly expressed in the cytoplasm and upon stimulation with the IFN, it accumulates in the cytoplasm (>1 × 10^6^ molecules per cell), and this abundance is identified to be crucial for its antiviral function^7^. Beside the distribution pattern of mouse IFIT1, no other IFIT proteins have been investigated for their subcellular distributions. To delineate the expression dynamics and sub-cellular distribution, DF-1 cells were transfected with V5-tagged chIFIT5 and transient expression of chIFIT5 was compared in NDV-stimulated or mock-treated cells. Confocal microscopy using anti-V5 antibodies showed that chIFIT5 was exclusively cytoplasmic and was expressed throughout the cytoplasm; however, this expression was concentrated at the cell surface (Fig. 1E, upper panel). We were unable to demonstrate the distribution pattern of chIFIT5 under NDV infection (Fig. 1E, lower panel) probably due to the profound antiviral state induced by chIFIT5 so that infection of NDV in the chIFIT5-expressing cells could not be achieved. It has previously been demonstrated that human IFIT5 enhances the innate immune responses by interacting with RIG-I (not identified in chicken) and MAVS and this interaction occurs at the mitochondria^11^. Since MAVS localizes on both mitochondria and endoplasmic reticulum (ER)-derived membranes (MEM), we labelled mitochondria and ER in presence of chIFIT5 to assess the distribution and localization of chIFIT5 with MAVS. In DF-1 cells, chIFIT5 localized in close proximity to the mitochondria and no co-localization was observed between ER and chIFIT5 (data not shown). Owing to lack of cross-reactivity of human IFIT5 antibodies and specificity of anti-sera which we have raised against chIFIT5, these experiments were performed using tagged-chIFIT5. Therefore, future studies are required to assess the expression patterns of the endogenous chIFIT5 under virus or non-virus stimuli.

### Transcriptional profiling of chIFIT5 *in vitro* and *ex vivo*

**Figure 2:**
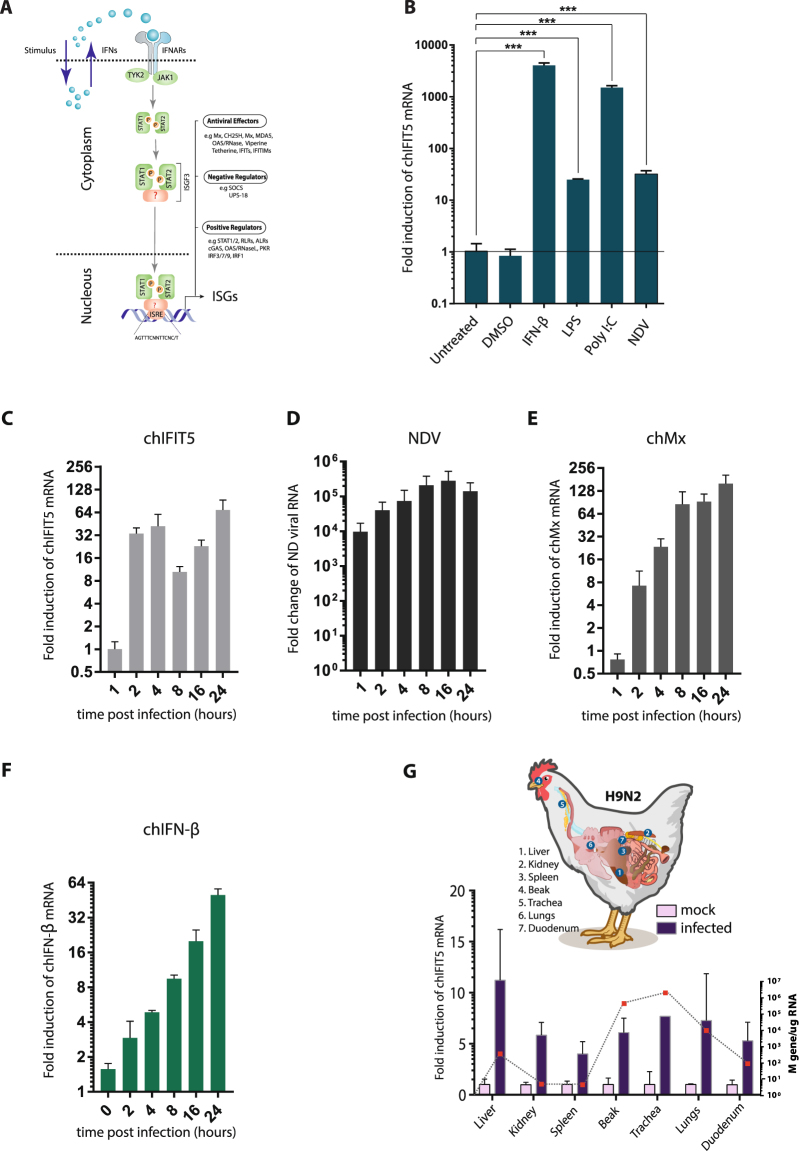
*In vitro* and *ex vivo* transcriptional regulation of chIFIT5 gene. (**A**) ChIFN-β itself, or produced by different stimuli, can initiate JAK-STAT signalling pathway and culminate in the induction of hundreds of ISGs. IFIT family of genes is one such antiviral effector of innate immune responses. (**B**) Quantitation of chIFIT5 mRNA in cells stimulated with 1000 U of chIFN-β, 10 μg/mL of LPS, 5 μg/mL of poly I:C or 1 MOI of NDV for 24 hours before RNA extraction and analysis for qRT-PCR using primers specific for the chIFIT5 gene. (**C–F**) Total cellular RNA was extracted from CEF cells at 1, 2, 4, 8, 16, and 24 hours post-NDV infection and was subjected to quantitation of mRNA for chIFIT5 (**C**), viral mRNA (**D**), chMx (**E**) and chIFN-β (**F**,**G**) RNA collected from seven organs infected or not with H9N2 influenza viruses were used to determine the level of chIFIT5 mRNA and M gene of the virus. Fold change induction in all experiments was determined by 2^−∆∆CT^ algorithm and data presented is average of three independent experiments.

We next assessed the nature of ligands that can transcriptionally induce the expression of chIFIT5. Different ligands that can either directly induce the transcription of IFIT5 such as IFN-β or stimuli that result in the expression of IFNs, which in turn induce the IFIT5 transcription, were assessed (Fig. 2A). TLR4 (lipopolysaccharide, LPS) and TLR3 (poly I:C, synthetic dsRNA) ligands significantly induced the expression of chIFIT5 gene by 25 and 1500 folds, respectively (Fig. 2B). The induction is likely through the activation of IFNs since the chIFN-β stimulated cells profoundly increased the transcription of chIFIT5 by 4000 folds compared to untreated or mock-treated cells. There are evidences that negative sense single stranded RNA viruses (e.g. influenza and NDV) produce dsRNA as intermediate by-product during the virus replication cycle^2,22–24^. Therefore, it is plausible that induction of chIFIT5 expression in chicken cells infected with NDV is mediated by virus-generated dsRNA (Fig. 2B). To further assess the temporal effect of NDV infection on the induction of chIFIT5, a time course profiling IFN and IFN-regulated genes was evaluated. The virus-induced expression of chIFIT5 was profoundly observed as early as 2 hours post-infection (Fig. 2C) and the expression was maintained for 4 hours. A slight reduction in IFIT5 expression was observed at 8 hours post-infection (hpi) was reconstituted at 24 hpi (Fig. 2C). This biphasic expression of IFIT5 after virus infection was repeatedly observed in chicken cells. However, the pattern of expression of myxovirus-resistance protein (Mx) gene, which is another well-characterized ISG^25^, shows a steady up-regulation and peak expression was observed at the latest time post-infection (Fig. 2E). The levels of chIFN-β gene induction was proportional to the NDV replication (Fig. 2D), therefore, it can be inferred that the virus-induced expression of chicken IFIT5 is IFN-dependent and that chIFIT5 is an early-ISG with capacity to modulate initial steps of virus life cycle.

To investigate transcriptional activation of IFT5 following virus infection in chicken, we analysed a panel of RNA transcripts derived from selected tissues (liver, kidney, spleen, beak, trachea, lungs and duodenum) of chickens (3-weeks-old Rhode Island Red) which were mock-infected or infected with H9N2 avian influenza virus strain A/Chicken/Pakistan/UDL01/08 (UDL08/H9N2). All tissues from infected birds contained relatively higher levels of chIFIT5 compared to the corresponding tissue samples derived from mock-infected chickens (Fig. 2G). These results conclude that virus infection positively regulates the transcriptional dynamics of chIFIT5 in chickens.

### Promoter structures contributing to the IFN, dsRNA or virus-mediated transcriptional activation of chIFIT5 gene

**Figure 3:**
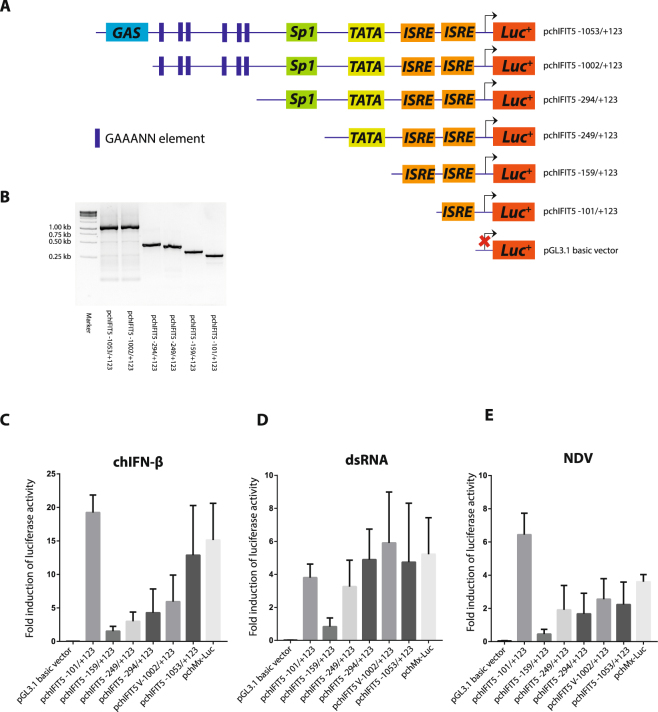
Promoter architecture and functional validation of chIFIT5 promoter responsiveness to chIFN-β, dsRNA and NDV. (**A**) Schematic presentation of full-length chIFIT5 promoter and subsequent construction of five truncated mutant constructs. (**B**) Shown are lengths of six total constructs upstream to firefly luciferase gene. (**C–E**) DF-1 cells were co-transfected with 150 ng of each of these chIFIT5 promoter-constructs along with 50 ng of pRenilla luciferase (loading control) for 24 hours and were stimulated with 1000 U/ml of chIFN-β (**C**), 5 ug/mL of dsRNA (**D**) or 1 MOI of NDV (**E**) for another 24 hours. Fold induction of firefly luciferase activities were measured and were normalized to Renilla luciferase. Data on viral and non-viral responsiveness of chIFIT5 promoter is average of three independent experiments.

Most studied-IFIT genes respond to IFN or IFN stimuli through one to four IFN-stimulated response elements (ISRE), which are mainly present within 200 base pair (bp) of the transcriptional start site in the orientation of the encoded gene or in the reverse complement order^6^. On the other hand, several IFIT genes lack ISRE motifs in their promoters including IFIT2 from chimpanzee, IFIT2B from human, chimpanzee and dog, and IFIT5 from horse, chimpanzee and dog^6^. Since our data and previously published transcriptomics studies^26^ support the profound expression of IFIT5 gene against a wide range of stimuli, we analysed the 5′ flanking region of chIFIT5 gene for motifs that regulate the expression of chIFIT5. In addition to the gene-encoding sequences, approximately 1.2 kb sequence including the putative promoter (Supplementary Fig. 2A) was isolated and sequenced. Inspection of the promoter region revealed the presence of two consecutive ISRE motifs within 85 bps from the transcriptional start site (Fig. 3A and Supplementary Fig. 2B). These elements were preceded by TATA element, and the binding motif for specificity protein 1 (Sp1) transcription factor. A putative and weak IFN-gamma-activated site (GAS) was identified at the distal end of the promoter and six consecutive GAAANN elements were predicted between GAS and Sp1 motifs (Fig. 3A). GAS is essential for IFN-gamma-mediated transcription of the genes whereas GAAANN elements have been demonstrated to regulate the virus-induced and type I IFN-regulated genes via the binding of interferon regulatory factors^27,28^.

To determine the role of these *cis-acting* regulatory elements in the responsiveness of different IFN ligands and stimuli, the entire promoter region (1122 bp) was cloned upstream to the luciferase gene in a promoter-less pGL3.1-Basic vector (Fig. 3A). Additionally, five reporter constructs were generated each containing either all of the GAAANN elements, Sp1, TATA box, double or single ISRE promoter sequences (Fig. 3A,B). Luciferase reporter assays were performed to assess whether the upstream region of the chIFIT5 gene can mediate responses to stimuli from chIFN-β, dsRNA or virus infection. DF-1 cells were transiently transfected with these reporter plasmids and subsequent luciferase activity was monitored after stimulation with either chIFN-β (Fig. 3C), dsRNA (Fig. 3D) or with NDV (Fig. 3E). Similar to IFN-induced promoter activation of chMx gene^25^, the chIFN-β induced 13-fold higher luciferase activities in full-length promoter (Fig. 3C). However, further deletion of GAS, GAAANN, Sp1 and TATA box resulted in an approximately 4-fold reduction in basal transcription activity. Additional deletion of the distal ISRE reduced the IFN-responsiveness of the promoter. However, repetitive luciferase assays demonstrated that ISRE motif just proximal to the transcriptional start site was exceedingly responsive to the IFN-treatment with a highest of 19-fold induction in luciferase activity as compared to the control vector without a promoter sequence (Fig. 3C). In the dsRNA-dependent promoter induction (Fig. 3E), results demonstrated that the above elements and motifs (GAAANN, SpI and TATA box) are least important in controlling the transcriptional activation of luciferase gene and a non-significant difference was observed in the absence or presence of these motifs. However, promoter sequence containing dual ISRE motifs severely compromised the responsiveness of dsRNA on the IFIT5 promoter and the dsRNA-dependent promoter induction was restored when distal-ISRE was deleted, further suggesting the importance of this ISRE motif (Fig. 3E). Similar to dsRNA induction, the construct containing only one ISRE motif was highly inducible by NDV compared to the construct carrying both ISRE motifs (Fig. 3E). As was observed in the NDV-induced chMx promoter activation, all other constructs with variable lengths and motifs were also responsive to virus infections. Comparison of these three stimuli indicated lower luciferase activities in NDV and dsRNA-treated chicken cells compared to chIFN-β induced promoter activation, presumably due to indirect activation of IFIT5 promoter by inducing endogenous IFNs.

**Figure 4:**
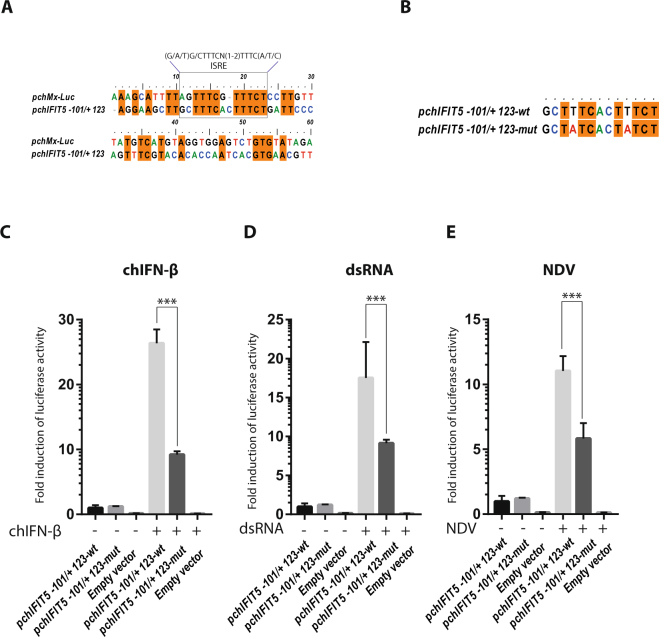
Identification and validation of functionally crucial motifs in chIFIT5 promoter immediately distal to the putative transcriptional start site. (**A**) Comparison of chMx and chIFIT5 responsive promoters, indicating a highly conserved ISRE element, which is composed of two triple-thymidines (TTT) motifs, which are separated by a single or double nucleotides in chMx and chIFIT5 promoters, respectively. (**B**) Mutagenesis of two triple thymidines motifs (TTT) to TAT in order to disrupt the core element are shown. (**C**–**E**) DF-1 cells were transfected with 150 ng of pchIFIT5–101/+123-wt, pchIFIT5–101/+123-mut promoter constructs or empty vectors along with 50 ng of pRenilla luciferase encoding loading control for 24 hours and were left unstimulated (−) or were stimulated (+) with 1000 U/ml of chIFN-β (**C**), 5 ug/mL of dsRNA (**D**) or 1 MOI of NDV (E) for another 24 hours before measuring the luciferase activities. Fold induction of firefly luciferase activities were measured and normalized to renilla luciferase and average data of three independent experiment is shown.

Following the demonstration of robust reporter function of the chIFIT5 promoter-containing proximal ISRE motif by the chIFN-β, dsRNA and NDV, we compared the sequence of *cis-acting* regulatory elements with the previously characterized chMx promoter^25^. *In silico* analysis of highly responsive 122 bp sequence indicated that the ISRE motif (5′-GCTTTCACTTTCT-3′ at position −29 to −13 in pchIFIT5 −101/+123 construct) was identical to the consensus ISRE (G/A/T)G/CTTTCN_1-2_TTTC(A/T/C) found in most of the IFN-inducible genes including chMx promoter sequence (Fig. 4A). For further absolute delineation of importance of this motif, we mutated both triplet thymidines (TTT), the core residues for the IFN-inducible activation of genes (Fig. 4B and Supplementary Fig. 2C). Responsiveness of the wild type (wt) and mutated ISRE motifs was assessed in chicken DF-1 cells with or without stimulation by chIFN-β, dsRNA or NDV using luciferase assays. While both pchIFIT5–101/+123 and pchIFIT5–101/+123-mut promoters were not auto-stimulatory, stimulation with either of the stimuli positively regulated the luciferase genes in pchIFIT5–101/+123. Interestingly, stimulation of mutated promoter with chIFN-β (Fig. 4C), dsRNA (Fig. 4D) or NDV (Fig. 4E) induced significantly lower promoter activation. These results revealed that the 16 nucleotide sequence containing single ISRE motif is crucial for the transcriptional regulation of the luciferase reporter gene. Taken together, structural and functional analysis of chIFIT5 promoter demonstrates that an ISRE motif approximately 16 nucleotides upstream of the transcription start site is minimum essential *cis-acting* element required for the transcriptional activation of the IFIT5 gene in chickens.

### Chicken and human IFIT5 restricts the replication of RNA virus in stably expressing chicken fibroblasts

**Figure 5:**
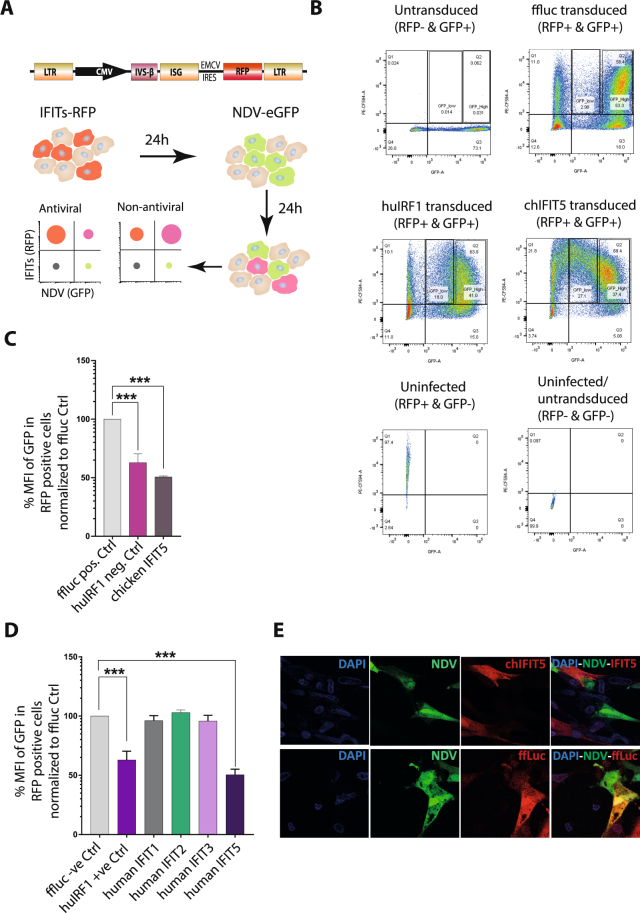
Stable overexpression of different IFIT proteins and their antiviral potential against NDV replication. (**A**) Lentiviruses bicistronically expressing the chIFIT5, huIRF1 or firefly luciferase (ffluc) along with RFP marker gene were transduced into the CEFs for 24 hours. These individual gene-expressing cell populations were then infected with NDV-GFP for another 24 hours before analysis using FACS. In the event of antiviral activities of the transgene, the percentage of GFP positivity would be reduced compared to ffluc control. (**B**) Cells were gated in untransduced (GFP+ and RFP−), uninfected (RFP+ and GFP−) or untransduced and uninfected (RFP- and GFP−) quadrants. Cells expressing ffluc, huIRF1 and chIFIT5 were then gated to each of these quadrants for GFP+ in RFP+ cell population. For demonstration purposes, GFP+ cell population was further divided into low GFP+ and high GFP+ cells indicating corresponding NDV replication. (**C**) Cumulative mean fluorescent intensity (MFI) of 5 independent experiments presented in section B showed significant inhibition of NDV in both huIRF1 and chIFIT5-transduced cells compared to ffluc control. (**D**) Similar to section B, cells were transduced with huIFIT1, huIFIT2, huIFIT3 and huIFIT5, and gated accordingly. Cumulative data of 3 independent experiments indicated a significant inhibition of virus replication in huIRF1 and huIFIT5 expressing cells. (**E**) CEFs were transduced with lentiviruses expressing ffluc or chIFIT5 and were infected with NDV-GFP. Individual cells co-expressing GFP and RFP were identified.

To assess the antiviral potential of chIFIT5 protein, we generated a lentivirus construct which bicistronically expresses chIFIT5 and a red fluorescent protein TagRFP under the control of EMCV (encephalomyocarditis virus) internal ribosome entry sequence (IRES)^3^. CEF cells were transduced with the appropriate VSV-G pseudotyped lentiviral particles and infected with GFP expressing NDV. After 24 hours, the virus infectivity (GFP+) was quantified in lentivirus transduced (RFP+) cell population using fluorescence-activated cell sorting (FACS) (Fig. 5A). Lentivirus expressing firefly luciferase (ffluc), not expected to affect the virus replication, was used as a negative control. Human IRF1 (huIRF1), a potent virus restriction factor^3^, was used as a positive control (Fig. 5B and Supplementary Fig. 3A). Compared to ffluc control, both huIRF1 and chIFIT5 significantly inhibited NDV replication (Fig. 5B,C), while the antiviral effect of chIFIT5 was greater than that of huIRF1 in CEFs. Since two populations of GFP+ cells were observed, further gating of low GFP+ and high GFP+ indicated a profound and cumulative antiviral effect compared to ffluc control (Supplementary Fig. 3B). These results showed that the chIFIT5-mediated antiviral effect is not profound at individual cell levels but it attenuates replication of the virus after initial infection.

To compare anti-viral effects of chIFIT5 (only IFIT gene identified in chickens so far) with its orthologous and homologous human proteins, huIFIT1, huIFIT2, huIFIT3 and huIFIT5 were expressed in chicken cells and antiviral affect was monitored (Fig. 5A). Similar to chIFIT5, both huIRF1 and huIFIT5 showed significant inhibition of NDV replication whereas no antiviral activities were observed by other huIFIT proteins in chickens (Fig. 5D). To further demonstrate the effect of IFIT5-induced inhibition of NDV replication, we observed an exclusive replication of either NDV (GFP+) or transduction of lentivirus expressing IFIT5 (RFP+) in 85% cells compared to ffluc expressing cells (*n* = 200) (Fig. 5E, upper panel). It is possible that transduction of lentivirus particles may interfere with the virus entry and/or NDV replication. To examine the lentivirus-mediated restriction of NDV replication, we used ffluc-transduced cells in the control group. The analysis of several microscopic fields showed simultaneous expression of lentiviruses-transduced ffluc protein and NDV infection (Fig. 5E, lower panel and Fig. 5B**)**, demonstrating that the transduction of lentivirus particles doesn’t interfere with the NDV replication. Collectively, these results demonstrate that IFIT5 proteins of both human and chicken are potent cellular restriction factors against RNA virus infection in chicken primary cells.

### CRISPR/Cas9 knockdown of chIFIT5 in chicken fibroblasts support the replication of viruses

**Figure 6:**
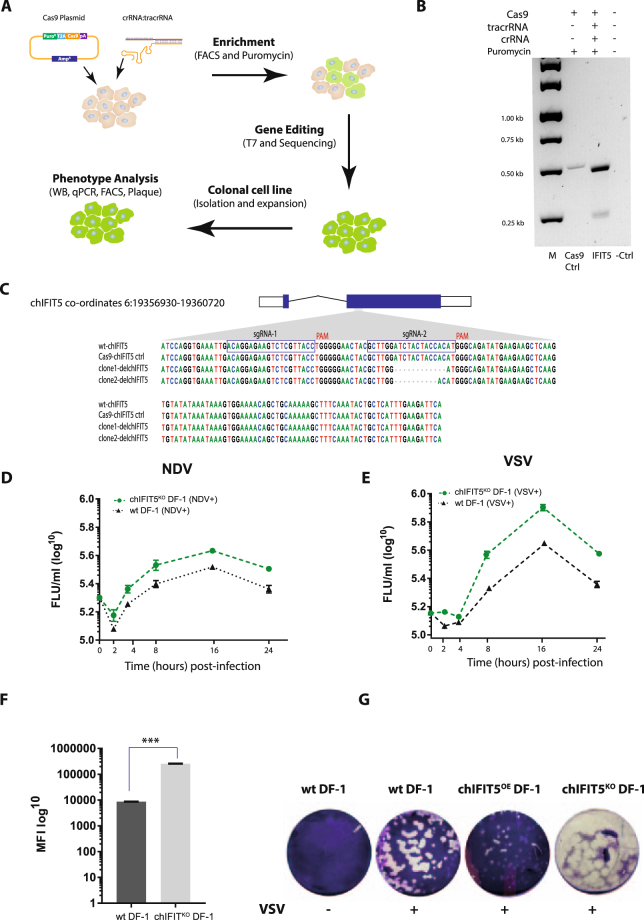
Knockout of chIFIT5 gene from chicken fibroblasts using CRISPR/Cas9 genome editing technology and evaluation of the influence of chIFIT5 on the replication of NDV and VSV. (**A**) DF-1 cells were co-transfected with hybridized crRNA and tracrRNA and plasmid expressing both Cas9 and puromycin resistance gene. 48 hours post-transfection; cells were passaged and selected with puromycin (10 ug/ml) until complete death of non-transfected cells. Puromycin-resistant cells were transfected with GFP expressing plasmid and were FACS sorted into individual cells and expanded until desire number of cells was achieved. At least 5 clones were confirmed for editing before clonal expansion and phenotypic analysis. (**B**) T7EI assays confirmation of the InDels in clonal cells that were supplemented with all essential components of gene editing including crRNA, tracrNA and Cas9 (lane number 3) compared to Cas9 control (lane number 2) and a no-input control (lane number 4). (**C**) Confirmation of gene editing using DNA sequencing of amplicons spanning the putative InDels site. (**D**,**E**) Wild type and chIFIT5^KO^-confirmed DF-1 cells were infected with NDV (**D**) or VSV (**E**). Replication of RNA viruses was monitored for 24 hours of post-infection and shown in fluorescent units. (**F**) Wild type and chIFIT5^KO^-confirmed DF-1 cells were infected with NDV or were left uninfected for 24 hours before processing them for FACS. Cumulative MFI of GFP positivity in wild type and chIFIT5^KO^ DF-1 cells based on at least 3 independent experiments. (**G**) DF-1 cells were either mock transfected (wt DF-1 cells) or were transfected with 2 ug of chIFIT5 expression plasmid (chIFIT5^OE^ DF-1 cells). These cells and CRISPR/Cas9 knockout DF1 (chIFIT5^KO^) were either left un-infected or were infected with VSV for 48 hours before staining with crystal violet. The plagues developments were imaged using hand-held camera.

To further evaluate the antiviral effect of chIFIT5 on virus replication, we applied a loss-of-function approach using CRISPR/Cas9 genome editing technology. Using synthetic gRNA and Cas9 expression plasmid (Fig. 6A), the chIFIT5 was targeted for editing. DF-1 cells were co-transfected with hybridized crRNA and tracrRNA as well as a vector expressing hSpCas9 and puromycin resistance marker (Puro^R^) gene. For fast and efficient enrichment of genetic modification, a population of stably-integrated cells was selected with puromycin, and was further enriched using FACS (Fig. 6A). The relative frequency of gene editing in the puromycin-resistant and FACS-enriched cell was estimated from a DNA mismatch detection assay using T7 Endonuclease I (T7E1) (Fig. 6B). T7E1 assay showed a mutation frequency of 40% within the chIFIT5 gene, however, T7E1 assays are likely to underestimate the fold enrichment^29^. We subsequently sequenced the mutated sites and confirmed the in-frame or out-of-frame gene editing (Fig. 6C). Results of the T7E1 assay and sequencing showed that sgRNA2 sufficiently edited the gene and puromycin selection greatly improved the enrichment of the cells. Additionally, most of the mutations in the chIFIT5 gene appeared to be deletions, which introduce a pre-mature stop codon to the beginning of Exon 2 of the chIFIT5 gene.

The chIFIT5 deletion-confirmed DF-1 cells (chIFIT5^KO^ DF-1) were isolated, expanded and assessed for virus replication. Both wt DF-1 and chIFIT5^KO^ DF-1 were infected with NDV and VSV, and replication of viruses was monitored for 24 hours. Both NDV (Fig. 6D) and VSV (Fig. 6E) replicated efficiently in wt DF-1 cells over the time course of infection (2 to 24 hours). However, deletion of chIFIT5 by CRISPR/Cas9 further supported the replication of NDV (Fig. 6D) and VSV (Fig. 6E), confirming that chIFIT5 is a crucial antiviral effector and elimination of such factors weakens the hostile barriers of the host. These results also highlight the possible exploitation of innate immune genes in promoting virus replication for vaccine production. Additionally, we applied FACS to quantify the percentage infectivity of the wt DF-1 and chIFIT5^KO^ DF-1 cells for GFP-expressing recombinant NDV. Data demonstrated that the wtDF-1 cells infectivity by NDV (45.6%) could further be enhanced (63.2%) in the absence of chIFIT5 (Supplementary Fig. 4). Cumulative quantitative measurement of NDV-infectivity was significantly enhanced in chIFIT5^KO^ DF-1 cells compared to wt DF-1 cells (Fig. 6F). Next, in order to counter-confirm the antiviral potential of chIFIT5, the level of VSV replication was assessed in conventional plague assay in DF1 cells either overexpressing or knocked out with chIFIT5 (Fig. 6G). The VSV replicated effectively in wt DF-1 cells and overexpression of chIFIT5 suppressed the VSV whereas CRISPR/Cas9 knockout DF-1 cells substantially supported the virus replication (Fig. 6G). Together, these results confirm the potential of chIFIT5 as an important host restriction factor, at least against evaluated RNA viruses.

### Chicken IFIT5 interacts with 5′ppp-containing RNA

**Figure 7:**
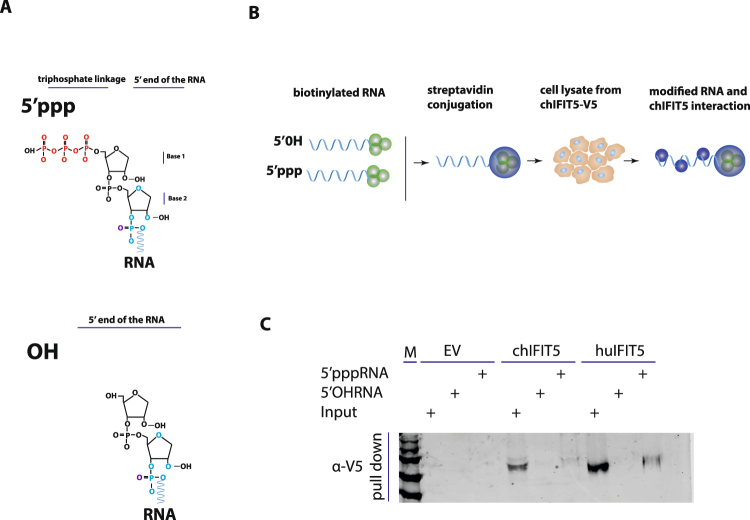
Interaction of chIFIT5 with RNA carrying modifications in their 5′ termini using RNA-protein immunoprecipitation. (**A**) The genomes of negative sense single stranded RNA viruses carry triphosphate linkage (5′ppp) in the first transcribed base of the RNA and removal of 5′ppp structure will leave non-IFN stimulatory hydroxyl group (OH). (**B**) RNA carrying both 5′ppp and OH termini were biotinylated and coated on streptavidin beads before interaction with V5-tagged chIFIT5. Total ribonucleoproteins were isolated and stained for the V5 tag. (**C**) Pull down of biotinylated RNA interacting with chIFIT5 indicated that both human and chicken IFIT5 interacted with RNA carrying 5′ppp structures. EV = empty vector, M = marker.

Different human and mouse IFIT proteins (IFIT1, IFIT2, IFIT3 and IFIT5) interact with RNA carrying multiple genetic modifications at their 5′ ends^9,19,30–32^. Since IFIT5 was the only identified IFIT protein in chickens, we aimed to explore whether chIFIT5 interacts with RNA in a similar mechanism reported for huIFIT5 or chIFIT5-RNA interaction is redundant to other members of IFIT family. In order to explore the molecular mechanisms of chIFIT5 recognition of RNA, we used 378 bp RNA without any known similarity to viral RNA sequences and structure, and generated (i) RNA bearing terminal 5′ hydroxyl group (5′OHRNA), and (ii) RNA bearing 5′ triphosphate (5′pppRNA) (Fig. 7A). These populations of RNA, mimicking viral RNA ends, were biotinylated and coupled with agarose beads. The beads were then incubated with chicken DF-1 cells expressing V5-tagged chIFIT5, the ribonucleoproteins were purified (Fig. 7B) and the interaction of chIFIT5 was determined by staining chIFIT5. While neither huIFIT5 nor chIFIT5 interacted with the 5′OHRNA, both proteins recognized RNA carrying ppp at the 5′ end (Fig. 7C).

### Overexpression of chicken chIFIT5 in transgenic chicken embryos restricts the replication of RNA virus

**Figure 8:**
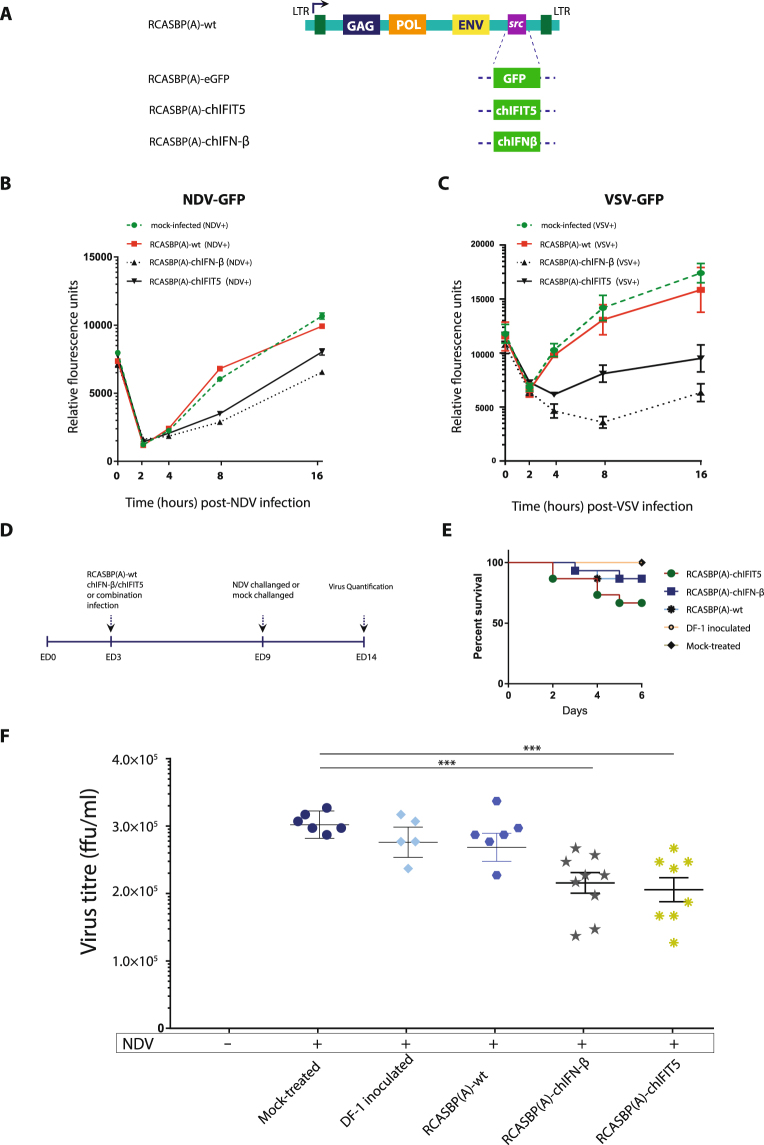
Generation of recombinant retroviruses and transgenic chicken embryos expressing marker gene (GFP), chIFN-β and chIFIT5, and their impacts on the replication of RNA viruses. (**A**) A schema for the generation of recombinant viruses in which *src* gene was replaced with either GFP, or chIFN-β or chIFIT5. (**B**,**C**) Stable DF-1 cells expressing chIFN-β (RCASA(BP)-chIFN-β) or chIFIT5 (RCASA(BP)-chIFIT5), DF-1 infected with wt retroviruses (RCASA(BP)-wt) or non-infectious DF1 cells (mock-infected) were infected with NDV (**B**) or with VSV (**C**) and the replication of viruses were monitored until 16 hours post-infection and data are presented in relative fluorescence units compared to non-infected background. (**D**) Infectious cells that imparted antiviral effects were inoculated in 3 days old chicken embryos and allowed to develop until 9 days post-embryonation when 100 PFUs of NDV were inoculated per transgenic egg and the quantification of the virus replication was performed on day 14 post-embryonation. (**E**) Survival curve in retrovirus-infected or mock-treated chicken embryos. (**F**) Quantitative analysis of viruses in NDV infected and un-infected transgenic embryos expressing indicated transgene. Each dot represents individual chicken embryo in all groups.

We next asked whether the *in vitro* demonstrated antiviral activities of chIFIT5 are translatable *in ovo* in developing chicken embryos. For this purpose, we constructed RCASBP(A)-chIFIT5 recombinant viruses to generate mosaic-transgenic chicken embryos that are constitutively expressing chIFIT5, and monitored its impact on the replication of NDV (Fig. 8A). In addition, a reporter virus, RCASBP(A)-eGFP, was generated to monitor the rescue of the virus and to investigate the effect of retroviruses on host responses. RCASBP(A)-chIFN-β, expressing chIFN-β which is a known antiviral cytokine^5^, was generated as a positive control (Fig. 8A). Expression of this transgene did not compromise the replication of retroviruses, and the induction of innate immune responses was not significant (data not shown), confirming previous reports that RCASBP is a safe *in ovo* gene transferring system^33,34^.

Stable cells lines expressing retrovirus-mediated chIFIT5 and chIFN-β were used to monitor virus replication. While mock-infected or RCASA(BP)-wt infected DF-1 cells did not interfere with the replication of NDV (Fig. 8B) and VSV (Fig. 8C), stable expression of chIFIT5 and chIFN-β established a profound antiviral state against both viruses over the time course of infection. These functionally validated infectious DF-1 cells were used to generate transgenic chicken embryos.

Three-day-old embryonated chicken eggs were inoculated with wt retroviruses or viruses that were expressing chIFIT5 or chIFN-β to generate mosaic transgenic chicken embryos (Fig. 8D). Additionally, embryos were either mock-inoculated with PBS or with non-infectious DF-1 cells to exclude the possibility of antiviral effects by the retrovirus infected DF-1 cells. These five groups of embryonated eggs were either mock-challenged with PBS or virus-challenged with NDV, and were incubated for another five days (Fig. 8D). Compared to controls, retroviral expression of IFIT5 negatively affected the development of healthy embryos until day 9 post-embryonation before challenge with NDV (Fig. 8E). Quantitative analysis of NDV in allantoic fluids showed that the wt retroviruses or non-infectious DF-1 cells alone were unable to interfere with the replication of NDV, however, transgenic embryos stably expressing chIFIT5 or chIFN-β significantly restricted the virus replication compared to the mock-treated control (Fig. 8F), indicating that chIFIT5 can restrict virus replication in developing transgenic chicken embryos. Collectively, these results further confirm the antiviral activity of chIFIT5 *in ovo*.

### Ablation of chicken chIFIT5 in transgenic chicken embryos ameliorates the replication of RNA viruses

**Figure 9:**
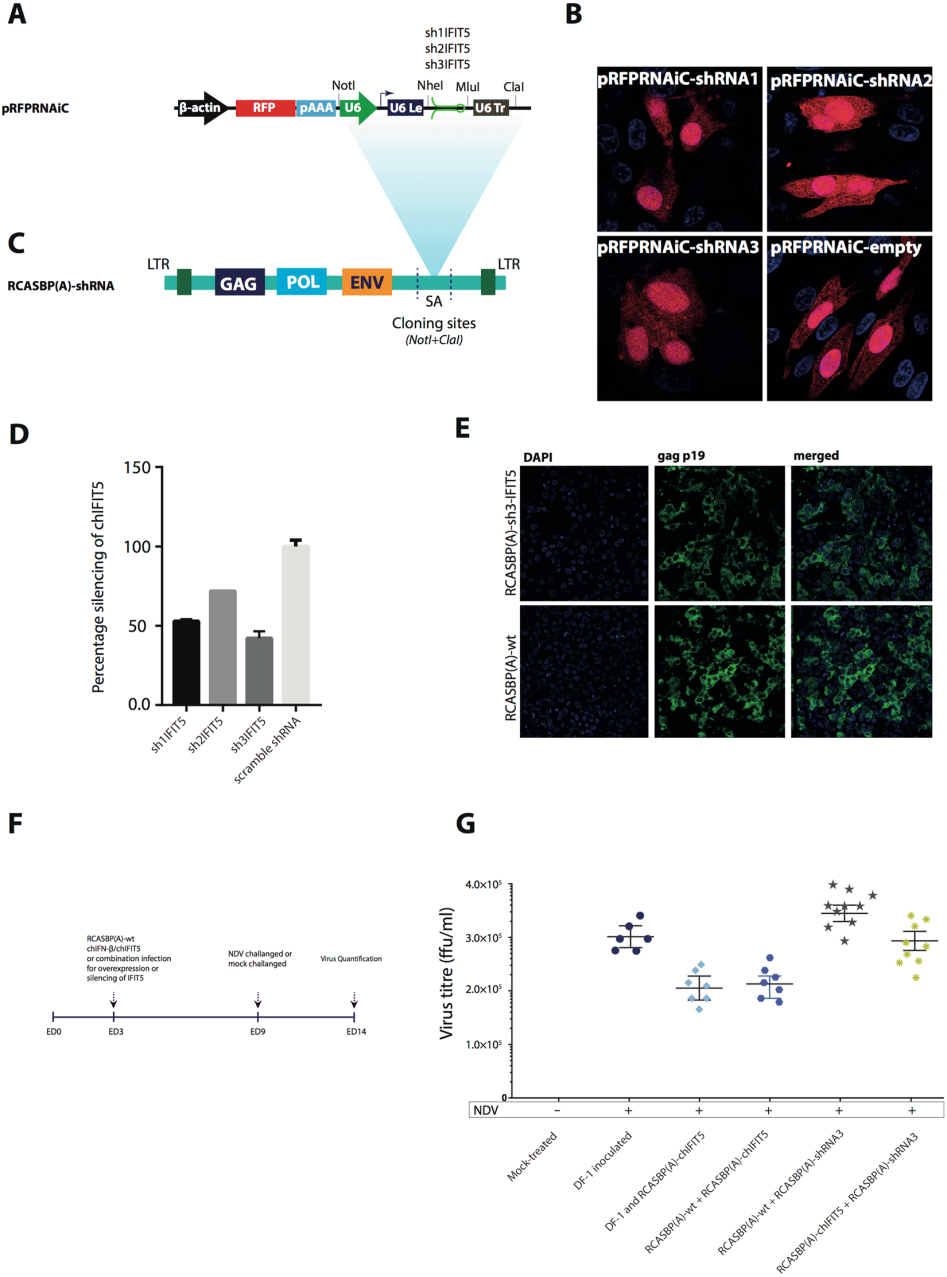
Silencing of endogenous chIFIT5 gene in transgenic chicken embryos and impact on the virus replication. (**A**) Three shRNA targeting exon 2 of chIFIT5 were cloned in pRFPRNAiC vector between NheI and MluI downstream to the chicken U6 promoter. (**B**) DF-1 cells were transfected with 500 ng of pRFPRNAiC-shRNA plasmid expressing each of the cloned shRNA or empty vector. The expression of RFP marker gene demonstrates the integrity of the constructs. (**C**) Transfer of validated shRNA (shRNA #3) cassette between NotI and ClaI sites in RCASBP(A) vector. (**D**) Silencing efficacies of all three chIFIT5-targeted shRNA compared to scrambled (non-targeting) shRNA. DF-1 cells, transfected with 500 ng of each of the plasmids, were used to extract total cellular RNA and the level of chIFIT5 gene silencing was monitored using qRT-PCR. (**E**) Retroviruses were rescued in DF-1 cells and stained for structural gag protein indicating the replication-competency of these retroviruses. (**F**) Infectious cells stably expressing pre-validated shRNA against chIFIT5 gene were inoculated in 3 days old chicken embryos and allowed to develop until 9 days post-embryonation when 100 FPUs of NDV were inoculated per transgenic egg and the quantification of the virus replication was performed on day 14 post-embryonation. (**G**) Quantitative analysis of viruses in NDV-infected and un-infected transgenic embryos expressing shRNA, wt chIFIT5 or shRNA-resistant chIFIT5. Each dot represents individual chicken embryo in all experimental groups.

Next, we asked whether silencing of endogenous chIFIT5 in developing embryos would facilitate the replication of NDV. For this purpose, we streamlined two individual gene delivery protocols for specific and optimal gene silencing in chicken cells and embryos. First, a total of three short hairpin RNAs (shRNA) targeting the chIFIT5 and a scrambled shRNA with a highly confident predicted score were cloned in the pRFPRNAiC as described before^35^ (Fig. 9A). Transfection of chicken DF-1 cells with these vectors expressing shRNA under a chicken U6 promoter showed a reliable level of expression of the RFP marker gene indicating the functional integrity of the system (Fig. 9B). Although all shRNA were effective in silencing, quantitative analysis of NDV-induced chIFIT5 mRNA showed that shRNA#3 was the most effective (>50%) (Fig. 9D). Next, the validated shRNA cassette that included chicken U6 promoter, shRNA, leader and trailer sequences was transferred to the compatible RCASBP(A) vectors for silencing of chIFIT5 *in ovo* (Fig. 9C). Additionally, we used shRNA silencing-resistant RCASBP(A)-chIFIT5 retroviruses (Supplementary Fig. 5) to stably express the chIFIT5 in developing embryos in the presence of shRNA against endogenous chIFIT5 gene. The replication competency of shRNA expressing retroviruses was compared to the wt retroviruses (Fig. 9E) before using them to generate transgenic chicken embryos.

Using the experimental strategy depicted in Fig. 9F, we monitored the replication of NDV in transgenic embryos that were either stably expressing chIFIT5 or were expressing chIFIT5 in the endogenously silenced IFIT5 gene. The retroviral overexpression of chIFIT5 or silencing of chIFIT5 were normalized to non-infectious DF-1 cells or wt RCASPB(A) that did not express the transgene. Quantitative analysis of NDV showed an expected reduction in replication of the virus in IFIT5-overexpressing embryos, whereas silencing of endogenous chIFIT5 favoured the replication of NDV compared to the control DF-1 cells (Fig. 9G). A moderate reduction in the virus replication was observed in transgenic embryos that were simultaneously silenced for endogenous chIFIT5 and were stably expressing silence-resistant chIFIT5 (Fig. 9G). Taken together, these results not only confirm the antiviral activities of chIFIT5 in different experimental settings but also highlight the potential use of RCASPB(A) in enhancing the replication of avian viruses in embryonated eggs.

## Discussion

Innate immune responses are key to the success of host resistance to virus infection and ISGs provide a front-line role in these defences by acting at multiple stages of the virus replication cycle^2–5^. Among the myriad of ISGs, members of the IFIT family have attracted recent attention for both immunological as well as virological reasons due to their abundant and profound transcription and translation responses to diverse stimuli such as IFNs and viruses^9,19^. Significant advances have been made during studies of the mechanisms of both human and mouse IFIT proteins^19^. However, the knowledge on the breadth and plasticity of the antiviral properties of IFIT proteins are currently inadequate for non-mammalian hosts. Characterizing the repertoire of IFIT proteins and investigating their functions in chicken are of special interest because of the unique features of the chicken immune system including the absence of essential components of innate immune induction and signalling such as RIG-I, IRF3, and IRF9^5^ in chickens. Intriguingly, while chickens are lacking these components, they still mount profound innate responses against virus infections. Understanding the alternative means of innate immune regulation and antiviral defences in chicken could establish the foundation to control chicken-mediated emergence of zoonotic infections such as influenza viruses^36^.

In order to explore the IFIT genes in chicken, we began with genetics and functional genomic approaches and revealed that chicken encodes only a single IFIT gene compared to 4 genes in mammals (human and mice). Based on its sequence and structure similarities, and phylogenetic associations, this single chicken IFIT gene is classified as IFIT5 (chIFIT5). Currently the chicken genome is about 90% annotated and all chromosomes are correctly characterized^37^, however, the possibility of orthlogous and pseudogenes in the non-annotated part (10%) of the chicken genome cannot be excluded at this stage. With regard to the complex IFN pathways and a number of major missing genes in chicken, the innate immune genes, as general sensors of self and non-self, are under continues evolutionary selection pressure compared to the pathogen-specific adaptive immune responses. Therefore, it is plausible that IFIT proteins are generated by paralogue expansions and/or gene deletions in chicken^6^. IFIT5 is the only gene that is missing in the mice and rat genomes^6^ whereas it is the only gene identified in chicken, as this study shows. It remains to be determined in future if chIFIT5 also plays additional antiviral functions of the redundant IFIT1 and IFIT3.

We observed a profound transcription of chIFIT5 gene by diverse stimuli that acted upon the IFN induction or IFN signalling pathways, which was further confirmed by the transcriptomics profiling of the MDV-infected chicken fibroblasts. Interestingly, structural and functional characterization of the chIFIT5 promoter, required for effective transcriptional regulation of chIFIT5, was mapped within 100 bp of the transcriptional start site and carried a single ISRE motif. This minimum essential requirement of the promoter justifies several folds induction of chIFIT5 gene as early as 2 hours of post NDV-infection. Although transcription of IFIT5 was generally proportional to the virus replication and IFN induction, an IFN-independent regulation of chIFIT5 is also possible, especially when the chIFIT5 gene up-regulation was observed in earlier time points of virus-infection when the IFN gene was barely detectable. It is also plausible that transcriptional activation of chIFIT5 is directly regulated by IRF7 or related transcription factors and thus warrants future investigations. Nevertheless, these transcriptional patterns augment essential roles of the chIFIT5 protein during the early stages of virus replication such as interaction with viral genetic material (viral RNA/DNA) and viral protein translation.

Previous RNA-protein analysis revealed that all IFIT proteins assemble into multimeric complexes (except IFIT5) and that these interactions are crucial for co-functionalities of these proteins^7^. While all IFIT proteins can make multimers, IFIT5 exists as a poorly characterized monomer^31^. Probably due to these structural flexibilities, reports on IFIT5 functionalities are inconsistent^7,30–32^. However, IFIT5 protein can arguably sense a broad range of RNA structures including single stranded RNA with mono-(p) and tri-phosphates (ppp) at the 5′ ends, double stranded DNA and RNA with CAP0 modifications^8,13,18^. In order to understand the binding potential of chIFIT5 to modified RNA that either interacts specifically with human IFIT5 or with human IFIT1, we coupled modified RNA-coated beads with quantitative binding assays for chIFIT5. Our results indicated that chIFIT5 specifically interacted with RNA that carried 5′-ppp modifications and failed to interact with RNA in which 5′-ppp was replaced with OH. These RNA-protein interaction studies highlighted the principal roles of chIFIT5 for direct recognition of foreign ppp-RNA and to subsequently exert downstream antiviral activities.

Since ppp-RNA is found within the genome of most viruses carrying negative sense single stranded RNA genomes such as influenza, NDV, VSV^38^, and ppp-RNA is produced as an intermediate product during the replication of viruses with positive sense RNA genomes such as coronaviruses^39^, it is plausible that chIFIT5 sense foreign RNA (bearing ppp-RNA) while ignoring self RNA (bearing CAP in the case of mRNA and monophosphate in the case of rRNA and tRNA). Compared to four essential ssRNA cellular sensors including RIG-I, TLR7, TLR8 and IFIT5 in mammals^1,13^, RIG-1 is missing and TLR8 is disrupted due to insertion of retroviruses in the TLR locus in chickens^5,39,40^. Because of these fundamental differences in innate immune genes between avian (e.g. chicken) and mammals, future studies are needed to investigate if the interaction of IFIT5 with 5′-ppp-ssRNA can induce downstream IFN signalling^11^, and if so, does this interaction compensate for the deficiency of RIG-I and TLR8 in chickens.

This specific interaction with ppp-RNA could lead to attenuation of virus replication by sequestering viral RNA for transcription and translation. To investigate this possibility, we applied both gain-in-function and loss-in-function methodologies and evaluated the antiviral potential of IFIT5 against negative sense single stranded RNA viruses, including a poultry specific (NDV) and model RNA virus (VSV). Lentivirus-delivered stable expression of chIFIT5 or huIFIT5 compromised the replication of viruses, whereas CRISPR/Cas9 mediated knocking-out of the chIFIT5 gene supported virus replication in chicken fibroblasts. Intriguingly, overexpression of human IFIT1, IFIT2 and IFIT3 failed to establish an antiviral state in chicken fibroblasts, suggesting that chickens have opted exclusively for the antiviral activities of IFIT5.

Our attempts to monitor virus replication in mosaic transgenic chickens overexpressing chIFIT5, or silenced for endogenous chIFIT5 yielded strong evidence that this cytokine possesses antiviral activities in developing embryos. Thus RCAS-mediated gene delivering and silencing approaches can be exploited to study gene functionalities *in ovo* at the early embryonic developmental stage and may establish the basis for evaluation of genetic resistance against pathogens.

Taken together, we characterized the IFIT locus in chicken and systemically analysed the functional rationale for antiviral activities of chIFIT5 against RNA viruses using both functional genomics and molecular biological approaches. The foundations built in this study warrant future investigations to assess the potential of chIFIT5 in sensing the nucleic acid of many diverse viruses and bacteria (which also generate pppRNA), and the impact of these interactions on host innate immunity.

## Materials and Methods

### Data mining and sequence analysis

Chicken genome (Ensembl) and expressed sequence tags (EST) databases were screened for the homologues of IFIT family gene members using the Basic Local Alignment Search Tool (BLAST) algorithm. A single transcript showing high sequence-similarity to human IFIT5 was identified in the putative IFIT locus. Using sequences from public databases and transcriptomics data from Marek’s disease virus (MDV)-infected chicken embryo fibroblasts (CEF), an open reading frame (ORF) was revealed and extracted. The chicken IFIT5 (chIFIT5) coding region was amplified from NDV-infected primary CEFs, whereas sequence-covering gap in the IFIT locus was amplified from genomic DNA using primers mentioned in Supplementary Table 1. The genomic nucleotide sequence of chIFIT5 promoter region was amplified using primers provided in Supplementary Table 1. The ORF and homology searches for chIFIT5 were carried out in the ORF Finder programme (http://www.ncbi.nlm.nih.gov/projects/gorf) and BLAST tool (http://www.ncbi.nlm.nih.gov/BLAST/) integrated in the NCBI database. Possible gene transcription start sites were identified using promoter predictor programme (http://www.fruitfly.org/seq_tools/promoter.html) whereas potential transcription factor binding sites were identified using the MatInspector server (http://www.genomatix.de). Gene synteny and tetratricopeptide repeats (TPR) were predicted using Ensemble as well as Conserved Domain Databases, respectively. The IFIT5 sequences from aves and non-aves were acquired from NCBI and aligned using ClustalW programme. The phylogenetic analysis was performed using neighbour-joining method with bootstrap value of *n* = 2,000 in the MEGA programme version 6^41^.

### Cells culture, media and antibodies

CEFs were prepared from 9-day-old embryonated eggs at The Pirbright Institute as described previously^42^. Immortalized chicken fibroblasts (DF-1), human embryonic kidney cells 293T (HEK-293T) and Madin-Darby canine kidney (MDCK) cells were maintained in Dulbecco’s modified eagle medium (DMEM) supplemented with 10% foetal bovine serum (FBS), 1% penicillin and streptomycin (P/S) at 37 °C in 5% CO_2_ incubator. AMV-3C2-S (gag) antibodies were purchased from Hybridoma Bank of Iowa, University of Iowa. α-V5 and J2 antibodies for the detection of V5 tag and dsRNA were from Genetex, and SCICONS, respectively. Alexa-flour 568 secondary antibodies were purchased from Invitrogen Carlsbad, CA, USA and IRDye 800CW α-mouse secondary antibodies were acquired from LI-COR, Nebraska USA. Poly I:C (a synthetic analogoue of dsRNA), dimethyl sulfoxide (DMSO) and lipopolysaccharide (LPS), were purchased from Invivogen and Sigma whereas chicken IFN-β was produced in HEK-293T cells^43^.

### Virus infections and quantifications

LaSota strain of NDV expressing green florescent protein (GFP) (NDV-GFP) was generated using reverse genetics system as described before^44^ and rescued virus particles were propagated in embryonated chicken eggs^45^. The NDV-GFP strain was quantified using immunostaining and expressed as focus-forming units (FFU). Vesicular stomatitis virus (VSV) expressing GFP (VSV-GFP) was kindly provided by Dennis Rubbenstroth (Institute for Virology, Medical Centre – University of Freiburg, Germany). VSV-GFP was propagated and quantified in DF-1 cells and was represented in FFU or images showing plaques. Allantoic fluids and infectious viruses from cell culture supernatants were titrated by plaque assays on MDCK cells. Briefly, MDCK cells were inoculated with 10-fold serially diluted samples and overlaid with 0.6% agarose (Oxoid, Hampshire, UK) in overlay DMEM (1× MEM, 0.21% BSA V, 1 mM l-glutamate, 0.15% sodium bicarbonate, 10 mM HEPES, 1× penicillin/streptomycin (Gibco, Carlsbad, CA, USA) and 0.01% Dextran DEAE, with 2 µg ml^−1^ TPCK trypsin (Sigma-Aldrich, Dorset, UK). Plates were incubated at 37 °C for 72 h and plaques were developed using crystal violet stain containing methanol.

### Plasmids construction and mutagenesis

The full-length chicken and human IFIT5 was PCR-amplified using primers that were tailed with 5′ BamHI site, 3′ EcoRI/SpeI site and a consensus Kozak translation sequence (CCACCATG) (Supplementary Table 1). The BamHI and EcoRI/SpeI digested amplicons were sub-cloned in the mammalian expression vector, pEFPLINK-V5 (kindly provided by Steve Goodbourn, St. George’s University of London), which contains an N-terminal V5 tag. For identification of *cis-acting* elements in the chIFIT5 promoter, a 1.2 kb genomic sequence was amplified and cloned into the KpnI and Xhol sites in the promoter-less vector, pGL3.1 basic (Promega) and named as pchIFIT5–1053/+123. Subsequent five truncated versions of the promoter were amplified from full-length pchIFIT5–1053/+123 using primers mentioned in the Supplementary Table 1 and cloned between KpnI and Xhol sites in the pGL3.1 basic vector. For production of the chIFN-β, the ORF for chIFN-β (Accession Number, NM_001024836) was cloned in pcDNA3.1+ and final constructs were labelled as pcDNA3.1-chIFN-β^43^. The reporter plasmid pGL3-p-chMx-luc was kindly provided by Nicolas Ruggli, Switzerland and Renilla luciferase plasmid (phRL-SV40) was purchased from Promega, Madison, WI, USA. Mitochondrial (DsRed2-Mito-7, #55838)) and ER (mCherry-ER-3, #55041) markers were obtained from Addgene. Triple thymidine duplex (TTTNNNTTT) in pchIFIT5–101/+123-wt construct was mutated into TATNNNTAT using QuikChange Lightning Site-Directed Mutagenesis Kit (Agilent Technologies) and was named as pchIFIT5–101/+123-mut. All mutagenesis oligonucleotides were designed in the QuikChange Primer Design Tool and these primers are provided in the Supplementary Table 1. All clones were sequenced from both ends for correct frame and orientation or were digested with unique cleavage sites to confirm the gene inserts.

### Confocal microscopy

Chicken cells were transfected with individual or combined plasmids for indicated time points using Lipofectamine 2000 (Invitrogen) at a ratio of 1:3 or were infected with lentiviruses, retroviruses or NDV-GFP for indicated time points. These cells were then fixed for 1 h in 4% paraformaldehyde and permeabilized using 0.1% Triton-X100 before incubation with primary antibodies raised against V5 tag, dsRNA (J2), or *gag* protein of retroviruses. Additionally, depending upon the experimental needs, different fluorescent markers (RFP, GFP) were used. Afterwards, cells were incubated with corresponding secondary antibodies at 37 °C for 2 h. After brief staining with 4′,6-diamidino-2-phenylindole (DAP1) (nuclear), slides were visualized using a Leica SP5 confocal laser scanning microscope.

### Western blotting

All the transfections for subsequent Western blot analysis were performed following the same protocol as described for immunofluorescence unless otherwise indicated. Cells were lysed in a hypotonic buffer and protease inhibitor cocktail (Sigma). Proteins were separated by SDS-PAGE under reducing conditions and analysed by Western blotting using anti-V5 (GenTek) and IRDye-labelled secondary antibodies (Li-Cor Biosciences). The signals were acquired and quantified using the Odyssey infrared imaging system (Li-Cor Biosciences).

### IFN bioassay

IFN-induced protection against VSV-GFP was used to identify IFN-producing stable clones and to quantify IFN preparations, as described before^46^. Briefly, DF-1 cells were seeded in 96-well plates until they are 90% confluent and treated with serial dilutions of supernatants containing interferons for 24 hours. These Interferon stimulated cells were inoculated with VSV-GFP (MOI of 1). At 24 hours post-infection, VSV-GFP replication was correlated with the change in GFP fluorescence signal intensities using Luminometer (Promega, Madison, WI, USA). The percentage antiviral activity of IFNs were determined by comparing the percentage reduction of corrected GFP signal intensity (GFP signal intensity of IFN treated and virus infected wells minus background fluorescence signal intensity of uninfected control) with the mock treated and VSV-GFP-infected control wells. One unit (U) of IFN in the tested IFN preparations was defined as the volume containing 50% inhibitory activity against VSV-GFP. A total of 1000 Us of IFNs were used for stimulation of CEF or DF-1 cells.

### Construction and rescue of lentiviruses expressing chicken and human IFIT proteins

A bicistronic expression and Gateway-compatible destination vector (pTRIP.CMV.IVSb.GENE.ires.TagRFP) for lentiviruses was kindly provided by Charles Rice, The Rockefeller University, USA. To generate chIFIT5 entry clone, gene encoding chIFIT5 was PCR amplified with oligonucleotides (Supplementary Table 1) containing *attB* sites flanking gene-specific sequences. PCR products were purified over Qiagen columns (Qiagen) and cloned into pDONR (Invitrogen) with BP Clonase. BP Clonase reactions were transformed into Escherichia coli (Invitrogen), and colonies were screened by restriction digestion and sequencing. The gene sequences from pENTR clones were moved into pTRIP.CMV.IVSb.GENE.ires.TagRFP using LR Clonase II (Invitrogen) according to the manufacturer’s instructions. After LR reaction products transformation, one or two colonies for each construct were grown in 3 ml Luria-Bertani (LB) broth with ampicillin, and transfection-quality plasmid DNA was purified over anion-exchange columns (Qiagen). Lentivirus constructs expressing human IFIT1, IFIT2, IFIT3, IFIT5, IRF1 (positive control) and ffluc (negative control) were kindly provided by Charles Rice, The Rockefeller University, USA. All constructs were sequenced using primers provided in Supplementary Table 1 to confirm the gene insertion before rescuing lentiviruses.

### Generation and quantification of lentiviral pseudoparticles

HEK-293T cells were seeded in poly-lysine pre-coated plates with seeding density of 4 × 10^5^ per well of 6-wells plates and were co-transfected with gene expressing proviral DNA (huIFIT1, huIFIT2, huIFIT3, huIFIT5, chIFIT5, huIRF1, or ffluc), HIV-I gag-pol and VSV-G in a ratio of 1:0.8:0.2 using Lipofectamine 2000 (Invitrogen). Supernatants collected at 48 h and 72 h post-transfection were cleared by centrifugation (1500 rpm for 5 min) and were pooled and supplemented with 20 mM HEPES and 4 μg/ul polybrene (Sigma). For titration of lentiviral pseudoparticles, CEF cells (1 × 10^6^) were transduced with serial dilutions of individually rescued pseudoparticles for 24 hours. Trypsinized cells were fixed with 4% paraformaldehyde and processed for FACS for quantification of percentage RFP+ cells. The volume of the lentiviral pseudoparticles that infected 50% of CEF cells was used to transduce CEFs. Titrated lentiviral pseudoparticles were stored at −80 °C before use and the same stock of the virus was used for all experimentation.

### Lentiviral transduction and flow cytometry analysis of virus replication

CEF cells were transduced with 10 MOI of lentivirus-expressing specific gene in DMEM media containing 5% FBS, 20 mM HEPES and 4 μg/ml polybrene. Transduction was facilitated by centrifugation (1000 g for 1 h at 37 °C) and cells were incubated at 37 °C. A day later, cells were infected with GFP-tagged virus (NDV-GFP) at 0.5 MOI and the infection was stopped after 1 hr and replaced with fresh DMEM containing 5% FBS. After 24 hours of infections, cells were trypsinised and the cell suspensions were incubated with live dead marker (Near IR cat no: L10119) according to the manufacturer’s protocol. The cells were then fixed with 2% paraformaldehyde (PFA) for 30 min before analysing the cells by flow cytometry. As described before^47^, live and singlet cells were gated based on forward and side scatter, and four-quadrant plots were generated using the untransduced and uninfected (RFP negative and GFP negative), uninfected (RFP positive and GFP negative), and untransduced (RFP negative and GFP positive) cells. Analysis was carried out using FlowJo software applying the same gating and analysis strategies for all samples.

### Construction of IFIT5 knockout cell line using CRISPR/Cas9

A synthetic gene-targeting approach was applied to specifically knockout the chicken IFIT5 from the chicken embryo fibroblasts. For this purpose, two components (crRNA and tracrRNA) of single guide RNA (sgRNA) which are crucial for targeting specificity and scaffolding/binding ability for CRISPR associated protein 9 (Cas9) nuclease were synthesized by Dharmacon. Targeting the beginning of the second exon of chicken IFIT5, two individual CRISPR RNA (crRNA) were designed with the highest-predicted score and lowest off-target affects; sgRNA1 ACAGGAGAAGTCTCGTTACC and sgRNA2 GCTTGGATCTACTACCACAT. Using DharmaFECT Duo Transfection Reagents, DF-1 cells were co-transfected with individual crRNA and a common trans-activating crRNA (tracrRNA) as well as plasmid expressing Cas9 nuclease and puromycin resistance gene (Puro^R^) separated by self-cleavage T2A sequence. Cells were split after 48 hours transfection and selected for puromycin antibiotics (10 μg/ml) for one week or until complete eradication of non-transfected control cells. For fast and efficient enrichment of genetic modification, a population of cells with stable integration was enriched using FACS. For this purpose, puromycin-selected cells were transfected with GFP-expression plasmid and individual cells were sorted by FACS before being seeded in 96-well plates. At least 15 clones were expanded and the relative frequency of gene editing in the puromycin-resistant and FACS-enriched cells was estimated from a DNA mismatch detection assay using T7 Endonuclease I (T7E1) (NEB). The DNA fragments flanking the target editing sites were amplified from genomic DNA extracted by DNeasy Kits (Qiagen) using primers mentioned in Supplementary Table 1. A total of 200 ng of the PCR products were denatured at 95 °C and allowed to anneal gradually at room temperature to form heteroduplex DNA. The re-hybridized DNA was digested with T7EI and resolved in a 1.8% agarose gel to determine the gene editing efficiency. Additionally, the PCR products were sequenced using PCR-amplification primers and aligned with the corresponding wild-type genomic sequence to identify mutations, deletions and insertions. T7EI and sequence verified clones were used to monitor virus replication.

### Expression of cytokines and virus genes in different chicken tissues and cell cultures

Total RNA was extracted from IFN-β (1000 U), LPS (10 μg/mL), dsRNA (5 μg/ml), or NDV-stimulated (MOI of 1) DF1 or CEFs using TRIzol reagents (Invitrogen, Carlsbad, CA, USA). Additionally, organs were collected from specific pathogen free (SPF) chickens, which were infected or mock-treated (intranasally) for 2 days with 10^6^ pfu of A/chicken/Pakistan/UDL-01/2008 (H9N2). A total of 200 ng of RNA was used in PCR reactions using SuperScript® III Platinum® SYBR® Green One-Step qRT-PCR Kit (Invitrogen, Carlsbad, CA, USA). The abundance of specific mRNA was compared to the 28S rRNA (Supplementary Table 1) in the Applied Biosystems Prism 7500 system. The reaction was carried out in ABI 7500 light cycler using the following thermo profile; 50 °C for 5 minutes hold, 95 °C for 2 minutes hold, followed by 40 cycles of 95 °C for 3 seconds and 60 °C for 30 seconds. Melting curve was determined at 95 °C for 15 seconds, 60 °C for 1 minute, 95 °C for 15 seconds and 60 °C for 15 seconds. Primers for ISGs including chIFIT5 are provided in Supplementary Table 1. Primers specific for a conserved region of the influenza A and NDV matrix genes were used as described previously^48,49^.

### Luciferase assays

To determine responsiveness of chicken IFIT5 promoter to chicken IFN-β, dsRNA, and virus-stimulation, chicken fibroblasts were grown in 96-well plate format at 2 × 10^4^ to 4 × 10^4^ cells/well and were co-transfected with 10 ng/well of a plasmid constitutively expressing Renilla luciferase (phRL-SV40; Promega, Madison, WI, USA) and 150 ng/well of full length pchIFIT5–1053/+123 construct or one of 5 truncated pchIFIT5 promoters. In addition pGL3-P-chMx-luc was used as a positive control whereas pGL3.1 basic vector was used as a negative control. Correspondingly, DF-1 cells were co-transfected with phRL-SV40 and pchIFIT5–101/+123-wt or pchIFIT5–101/+123-mut constructs. All transfections were performed using Lipofectamine 2000. In all conditions, 24 hours post-transfection, cells were stimulated with either chIFN-β (1000 U), or dsRNA (5 μg/ml) or NDV (MOI = 1) or were left unstimulated. Cells were then lysed using 20 μl 1× passive lysis buffer (Promega, Madison, WI, USA), and samples were assayed for Firefly and Renilla luciferase activity using the Dual-luciferase Reporter Assay System by following supplier’s instructions using Luminometer (Promega, Madison, WI, USA).

### *In vitro* transcription of biotinylated synthetic RNA and modifications of 5 prime ends

Plasmid encoding antisense 7SK RNA (7SK-as) was linearized with unique BamHI restriction digestion and the purified DNA was used for *in vitro* transcription in the presence of bioin-16-UTP using RiboMAX™ Large Scale RNA Production System-SP6 (Promega, Cat# P1280) as recommended by the manufacturer and reported previously^17^ with a few modifications. Briefly, a reaction of 100 μl was established containing 20 μl 5 × SP6 buffer, 10 μl NTP-bioUTP mixtures, 5 μg linearized plasmid, and 10 μl enzyme mix. The reaction mixture was first incubated at 37 °C for 3 hours followed by digestion of the DNA remnant with 4U RNase-Free DNAse (Thermo Scientific) for another 30 minutes at 37 °C. The biotinylated uracil triphosphate (bioin-16-UTP) was incorporated during *in vitro* transcription for purification of ribonucleoproteins and due to nascent nature of polymerase a 5′-pppRNA was over-hanged as a signature for IFIT5 protein interaction. After the completion of *in vitro* transcription, the quality of *in vitro* transcription RNA was assessed by agarose gel electrophoresis and RNA was purified with RNeasy MinElute Cleanup Kit (Qiagen) according to the manufacturer’s recommendations. A total of 30 μg of purified *in vitro* transcribed and biotinylated ppp-RNA was either mock treated or dephosphorylated using alkaline phosphatase (FastAP, Fermentas) to remove 5′ triphosphate (ppp) which leaves an OH group (OH-RNA). RNA samples were purified with RNeasy MinElute Cleanup Kit and eluted with 40 μl nuclease-free water for use in the RNA-protein interactions.

### RNA-protein immunoprecipitation for identification of IFIT5-binding RNA

For purification of IFIT5-binding RNAs, streptavidin affinity resin was incubated for 60 minutes at 4 °C with 1 μg biotinylated OH-RNA or PPP-RNA in TAB buffer (50 mM Tris pH 7.5, 100 mM NaCl, 5% (v/v) glycerol, 0.2% (v/v) Nonidet-P40, 1.5 mM MgCl2) in the presence of protease inhibitor cocktail (EDTA-free, cOmplete; Roche) and RNAse inhibitor (Fermentas). To prepare chIFIT5 protein, chicken DF-1 cells (1 × 10^6^) were transfected with 5 μg V5-tagged chIFIT5 plasmid for 48 hours and lysed with TAP buffer in the presence of protease and RNAse inhibitors. The RNA-coated beads were incubated with 2 mg chIFIT5 protein lysate on a rotary wheel for 4 hours at 4 °C, and washed three times to remove unbound proteins. These beads were mixed with loading buffer and resolved on SDS before probing for anti-V5 primary antibodies with IRDye-labelled secondary antibodies (Li-Cor Biosciences). The signals were acquired and quantified using the Odyssey infrared imaging system (Li-Cor Biosciences).

### Short hairpin design and expression systems

To silence endogenous chIFIT5 gene in developing embryos, a total of three 22-nucleotides-long RNA interference (RNAi) short hairpin RNAs (shRNA) were designed using the Genscript RNAi target finder (https://www.genscript.com/ssl-bin/app/rnai). Double stranded DNA products for each of three chIFIT5 specific and a control scrambled target were generated by PCR using random and gene-specific oligonucleotides together with 1hp-L and 1hp-R (Supplementary Table 1) as described before^33^. The amplified PCR products were cloned between NheI and MluI into microRNA (miRNA) cloning sites of pRFPRNAiC (kind gift of Stuart Wilson, The University of Sheffield, UK). All shRNA coding plasmids were sequenced to confirm the inserts and orientations. To evaluate the silencing potential of individual shRNA, DF-1 cells were transfected with 500 ng plasmid using Lipofectamine 2000 according to the manufacturer’s protocol and the knockdown effects on the chicken IFIT5 was monitored and compared with the scrambled RNA transfected control. Next, the validated shRNA cassette that included chicken U6 promoter, individual shRNA, leader and trailer sequences was transferred to the RCASBP(A) retrovirus vector between NotI and ClaI sites. Rescue of RCASBP(A)-sh3IFIT5 retroviruses and generation of mosaic transgenic chickens are detailed below.

### Construction and rescue of IFIT5 expressing RCAS system, and generation of transgenic embryos

The ORF of chicken IFIT5 and coding regions (signal and mature peptide sequence) of the chIFN-β were amplified from RNA extracted from the NDV-infected primary CEFs. The amplified products were sub-cloned into an improved version of RCASBP(A)-ΔF1 (kindly provided by Stephen H. Hughes, National Cancer Institute, MD, USA) *via* the ClaI and MulI restriction sites which replace the *src* gene while maintaining the splice accepter signals. The resultant constructs were named as RCASBP(A)-chIFIT5 and RCASBP(A)-chIFN-β. Similarly, a GFP encoding RCASBP(A), referred as RCASBP(A)-eGFP, was generated by introducing the coding sequence of the GFP in between the ClaI and MulI sites. Additionally, a codon optimized and shRNA-silencing resistant chIFIT5 gene was chemically synthesized (GeneArt, Invitrogen) and cloned in the corresponding sites and labelled as RCASBP(A)-shRNA3. The inserted gene orientation and sequence validity were confirmed by DNA sequencing.

To rescue recombinant RCASBP(A) viruses, a total of 2.5 × 10^5^ DF-1 cells were seeded in 25 cm^2^ flasks and maintained at 37 °C, 5% (vol/vol) CO_2_ for 24 hours (~80% confluent). Cells were washed with PBS and transfected with 2.5 μg of each of the RCASBP(A)-wt, RCASBP(A)-eGFP, RCASBP(A)-chIFN-β and RCASBP(A)-chIFIT5 plasmids using Lipofectamine 2000 in OptiMEM with a pre-determined optimized ratio of 1:6 (Invitrogen, Carlsbad, CA, USA). Media were changed 6 hours post transfection and the cells were maintained in DMEM supplemented with 10% FCS and 1% penicillin/streptomycin for 48 hours. Expression of the reporter gene (GFP) was monitored using fluorescence microscopy whereas replication efficiencies of chIFN-β and chIFIT5 expressing retroviruses were assessed by staining the structural protein of RCASBP(A). The GFP/gag expression-confirmed cell cultures were split into 25 cm^2^ flasks and were passaged again into 75 cm^2^ flasks after 3 days. Finally cells were expanded into 150 cm^2^ flasks until the desired number (10^6^ cells/egg) was achieved.

Mosaic-transgenic chicken embryos were generated by inoculation of one million RCASBP(A)-chIFN-β, RCASBP(A)-chIFIT5, RCASBP(A)-shRNA3 and RCASBP(A)-wt infected DF-1 cells at day 3 post-embryonation or were left un-infected. At day 9 post-embryonation (6 days post-infection), each egg was either left unchallenged or was challenged with 100 PFU NDV-GFP. Embryo mortality was monitored daily and allantoic fluids were harvested at 14 days of embryonation and were subjected to the plaque assay for virus quantification.

### Statistical analysis

Pairwise comparisons of treated and control groups were performed using Student’s *t*-test. Multiple comparison of treatment groups were analysed using one-way analysis of variance (ANOVA). All statistical tests were conducted in the GraphPad Prism (GraphPad Software, La Jolla, CA, USA).

### Ethics Statement

All animal studies and procedures were carried out in strict accordance with the guidance and regulations of European and United Kingdom Home Office regulations under animal risk assessment numbers AR000789 and AR000791. As part of this process the work has undergone scrutiny and approval by the Ethics Committee at The Pirbright Institute.

## Electronic supplementary material

Supplementary Figures and tables

## References

[1] Randall RE, Goodbourn S (2008). Interferons and viruses: an interplay between induction, signalling, antiviral responses and virus countermeasures. Journal of General Virology.

[2] Goubau D, Deddouche S (2013). & Reis e Sousa, C. Cytosolic sensing of viruses. Immunity.

[3] Schoggins JW, Rice CM (2011). Interferon-stimulated genes and their antiviral effector functions. Curr Opin Virol.

[4] Pestka S, Langer JA, Zoon KC, Samuel CE (1987). Interferons and their actions. Annu Rev Biochem.

[5] Santhakumar, D., Rubbenstroth, D., Martinez-Sobrido, L. & Munir, M. Avian Interferons and Their Antiviral Effectors. *Front*. *Immunol*. **8**(2017). 10.3389/fimmu.2017.00049 PMC5281639 28197148

[6] Liu, Y., Zhang, Y. B., Liu, T. K. & Gui, J. F. Lineage-Specific Expansion of IFIT Gene Family: An Insight into Coevolution with IFN Gene Family. *Plos One* **8**(2013). 10.1371/journal.pone.0066859 PMC3688568 23818968

[7] Pichlmair A (2011). IFIT1 is an antiviral protein that recognizes 5′-triphosphate RNA. Nature Immunology.

[8] Abbas YM, Pichmair A, Gorna MW, Superti-Furga G, Nagar B (2013). Structural basis for viral 5′-PPP-RNA recognition by human IFIT proteins. Nature.

[9] Ganes S, Fensterl V (2015). Interferon-induced Ifit proteins: Their role in viral pathogenesis. Cytokine.

[10] Li Y (2009). ISG56 is a negative-feedback regulator of virus-triggered signaling and cellular antiviral response. Proceedings of the National Academy of Sciences of the United States of America.

[11] Zhang BH, Liu XY, Chen W, Chen L (2013). IFIT5 potentiates anti-viral response through enhancing innate immune signaling pathways. Acta Biochimica Et Biophysica Sinica.

[12] Katibah GE (2013). tRNA Binding, Structure, and Localization of the Human Interferon-Induced Protein IFIT5. Molecular Cell.

[13] Katibah GE (2014). Broad and adaptable RNA structure recognition by the human interferon-induced tetratricopeptide repeat protein IFIT5. Proceedings of the National Academy of Sciences of the United States of America.

[14] Decroly E, Ferron F, Lescar J (2012). & Canard, B. Conventional and unconventional mechanisms for capping viral mRNA. Nature Reviews Microbiology.

[15] Xiao S, Scott F, Fierke CA, Engelke DR (2002). Eukaryotic ribonuclease P: a plurality of ribonucleoprotein enzymes. Annu Rev Biochem.

[16] Leung DW, Amarasinghe GK (2016). When your cap matters: structural insights into self vs non-self recognition of 5′ RNA by immunomodulatory host proteins. Current Opinion in Structural Biology.

[17] Habjan, M. *et al*. Sequestration by IFIT1 Impairs Translation of 2′ O-unmethylated Capped RNA. *Plos Pathogens* **9** (2013). 10.1371/journal.ppat.1003663 PMC3789756 24098121

[18] Kumar P (2014). Inhibition of translation by IFIT family members is determined by their ability to interact selectively with the 5′-terminal regions of cap0-, cap1-and 5′ ppp- mRNAs. Nucleic Acids Research.

[19] Diamond MS, Farzan M (2013). The broad-spectrum antiviral functions of IFIT and IFITM proteins. Nature Reviews Immunology.

[20] Hughes SH (2004). The RCAS vector system. Folia Biol (Praha).

[21] Smith JB, Herschman HR (1996). The glucocorticoid attenuated response genes GARG-16, GARG-39, and GARG-49/IRG2 encode inducible proteins containing multiple tetratricopeptide repeat domains. Archives of Biochemistry and Biophysics.

[22] Jacobs BL, Langland JO (1996). When two strands are better than one: the mediators and modulators of the cellular responses to double-stranded RNA. Virology.

[23] Son KN, Liang Z, Lipton HL (2015). Double-Stranded RNA Is Detected by Immunofluorescence Analysis in RNA and DNA Virus Infections, Including Those by Negative-Stranded RNA Viruses. J Virol..

[24] Zhang S (2014). Activation of the PKR/eIF2α signaling cascade inhibits replication of Newcastle disease virus. Virol J..

[25] Schumacher B, Bernasconi D, Schultz U, Staeheli P (1994). The Chicken Mx-Promoter Contains an Isre Motif and Confers Interferon Inducibility to a Reporter Gene in Chick and Monkey Cells. Virology.

[26] Hui RK, Leung FC (2015). Differential Expression Profile of Chicken Embryo Fibroblast DF-1 Cells Infected with Cell-Adapted Infectious Bursal Disease Virus. PLoS One.

[27] Hiscott J (2007). Triggering the innate antiviral response through IRF-3 activation. J Biol Chem.

[28] Smale ST, Kadonaga JT (2003). The RNA polymerase II core promoter. Annu Rev Biochem.

[29] Kim, J. M., Kim, D., Kim, S. & Kim, J. S. Genotyping with CRISPR-Cas-derived RNA-guided endonucleases. *Nature Communications* **5**(2014). 10.1038/ncomms4157 24445736

[30] Terenzi F, Hui DJ, Merrick WC, Sen GC (2006). Distinct induction patterns and functions of two closely related interferon-inducible human genes, ISG54 and ISG56. Journal of Biological Chemistry.

[31] Feng F (2013). Crystal structure and nucleotide selectivity of human IFIT5/ISG58. Cell Research.

[32] Wacher C (2007). Coordinated regulation and widespread cellular expression of interferon-stimulated genes (ISG) ISG-49, ISG-54, and ISG-56 in the central nervous system after infection with distinct viruses. Journal of Virology.

[33] Kothlow S, Schenk-Weibhauser K, Ratcliffe MJ, Kaspers B (2010). Prolonged effect of BAFF on chicken B cell development revealed by RCAS retroviral gene transfer *in vivo*. Mol Immunol.

[34] Reuter A (2014). Antiviral activity of lambda interferon in chickens. J Virol.

[35] Das RM (2006). A robust system for RNA interference in the chicken using a modified microRNA operon. Developmental Biology.

[36] Barber MRW, Aldridge JR, Webster RG, Magor KE (2010). Association of RIG-I with innate immunity of ducks to influenza. Proceedings of the National Academy of Sciences of the United States of America.

[37] Schmid M (2015). Third Report on Chicken Genes and Chromosomes 2015. Cytogenetic and Genome Research.

[38] Kell AM, Gale M (2015). RIG-I in RNA virus recognition. Virology.

[39] Iwasaki A (2012). A virological view of innate immune recognition. Annu Rev Microbiol.

[40] Philbin VJ (2005). Identification and characterization of a functional, alternatively spliced Toll-like receptor 7 (TLR7) and genomic disruption of TLR8 in chickens. Immunology.

[41] Tamura K, Stecher G, Peterson D, Filipski A, Kumar S (2013). MEGA6: Molecular Evolutionary Genetics Analysis version 6.0. Mol Biol Evol.

[42] Schat, K. A. & Purchase, H. G. Cell-culture methods. In *A Laboratory Manual for the Isolation and Identification of Avian Pathogens*, Vol. 1 (eds Swayne, D.E., Glisson, J. R., Jackwood, M. W., Pearson, J. E. & Reed, V. W. M.) 223 (American Association of Avian Pathologists, Kennet Square, PA, 1998).

[43] Santhakumar, D., Nair, V.K., Iqbal, M. & Munir, M. Chicken IFN Kappa: A Novel Cytokine with Antiviral Activities. *Scientific Report* In Press (2017). 10.1038/s41598-017-02951-2 PMC5457445 28578423

[44] Al-Garib SO, Gielkens AL, Gruys E, Peeters BP, Koch G (2003). Tissue tropism in the chicken embryo of non-virulent and virulent Newcastle diseases strains that express green fluorescence protein. Avian Pathol.

[45] Munir M (2012). Biological characterization and phylogenetic analysis of a novel genetic group of Newcastle disease virus isolated from outbreaks in commercial poultry and from backyard poultry flocks in Pakistan. Infect Genet Evol.

[46] Berger Rentsch M, Zimmer G (2011). A vesicular stomatitis virus replicon-based bioassay for the rapid and sensitive determination of multi-species type I interferon. PLoS One.

[47] Karki S (2012). Multiple interferon stimulated genes synergize with the zinc finger antiviral protein to mediate anti-alphavirus activity. PLoS One.

[48] Wise MG (2004). Development of a real-time reverse-transcription PCR for detection of Newcastle disease virus RNA in clinical samples. Journal of Clinical Microbiology.

[49] Spackman E (2002). Development of a real-time reverse transcriptase PCR assay for type A influenza virus and the avian H5 and H7 hemagglutinin subtypes. Journal of Clinical Microbiology.

