# Supplementary material for: Chicken Interferon-induced Protein with Tetratricopeptide Repeats 5 Antagonizes Replication of RNA Viruses
Source: Sci Rep. 2018 May 1;8:6794. doi: 10.1038/s41598-018-24905-y (PMC5931624; doi:10.1038/s41598-018-24905-y)
Supplement: Supplementary file 1 — Supplementary Figures and tables [file 41598_2018_24905_MOESM1_ESM.pdf]

Supplementary Figures

Chicken Interferon-induced Protein with Tetratricopeptide Repeats 5 Antagonizes Replication of RNA Viruses

Diwakar Santhakumar[1,2¶], Mohammed Abdel Mohsen Shahaat Rohaim[1,2,3¶], Hussein A Hussein[3], Pippa Hawes[2], Helena Lage Ferreira[4], Shahriar Behboudi[2,5], Munir Iqbal[2], Venugopal Nair[2], Clarice W. Arns[6], Muhammad Munir[1,2*]

[1]*Division of Biomedical and Life Sciences, Faculty of Health and Medicine, Lancaster University, Lancaster LA1 4YG, UK,* [2]*The Pirbright Institute, Woking, Surrey, GU24 0NF, UK,* [3]*Faculty of Veterinary Medicine, Cairo University, Giza, 12211, Egypt,* [4]*Universidade de São Paulo, Campus de Pirassununga, Brazil,* [5]*Department of Pathology and Infectious Disease, School of Veterinary Medicine, University of Surrey, UK,* [6]*Institute of Biology, University of Campinas, Brazil*

*Corresponding Author: drmunir.muhammad@gmail.com; m.munir@lancaster.ac.uk

[¶] Authors contributed equally.

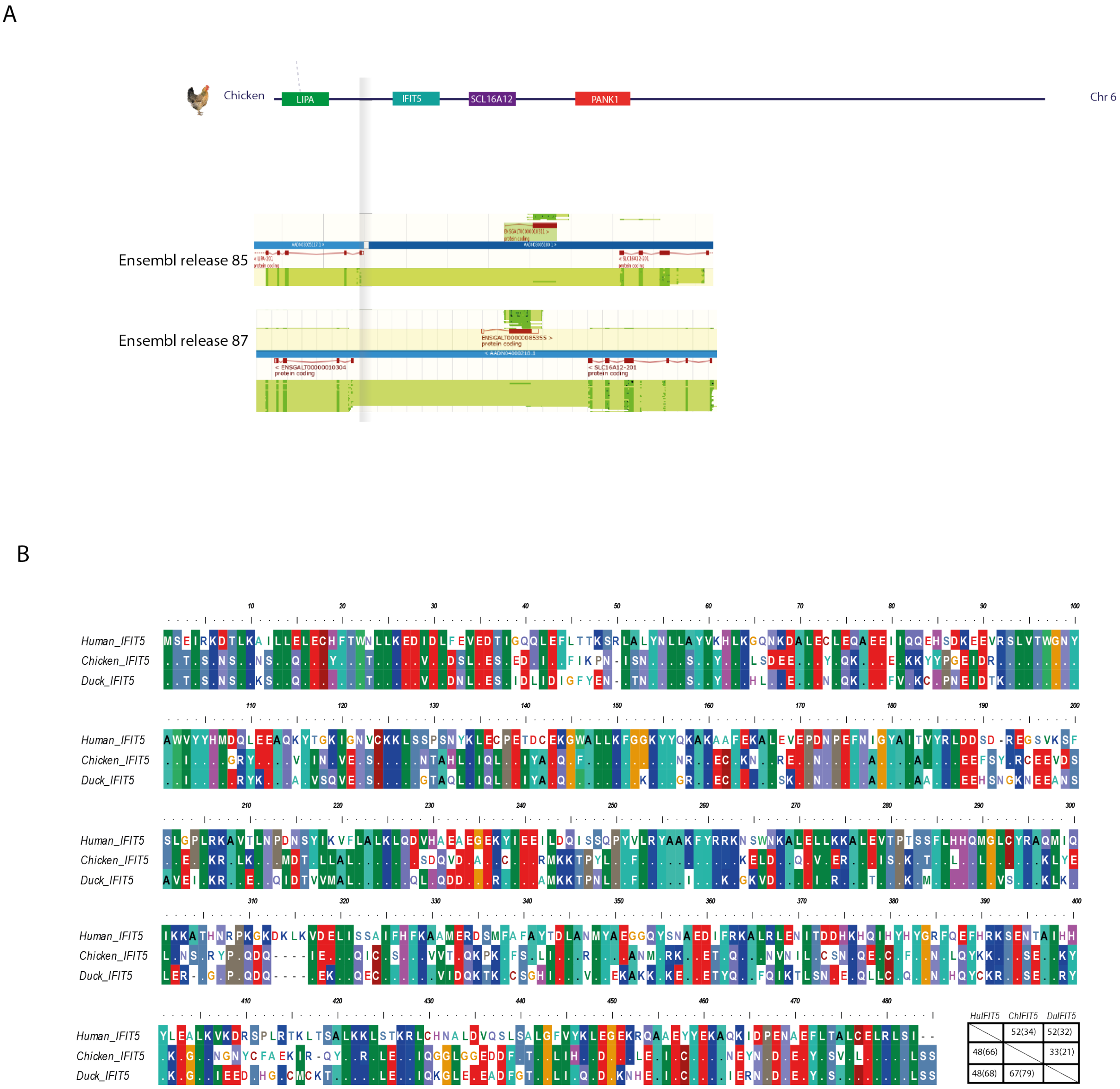

Figure S1│Bioinformatics analysis of chIFIT5 gene. (**a**) Pairwise sequence alignment of chicken, duck and human IFIT5 proteins. A comparative percentage positivity and negativity is shown in the box. (**b**) Comparison of IFIT locus in two Ensembl releases. The genomic gap downstream to LIPA gene is highlighted with vertical grey bar.

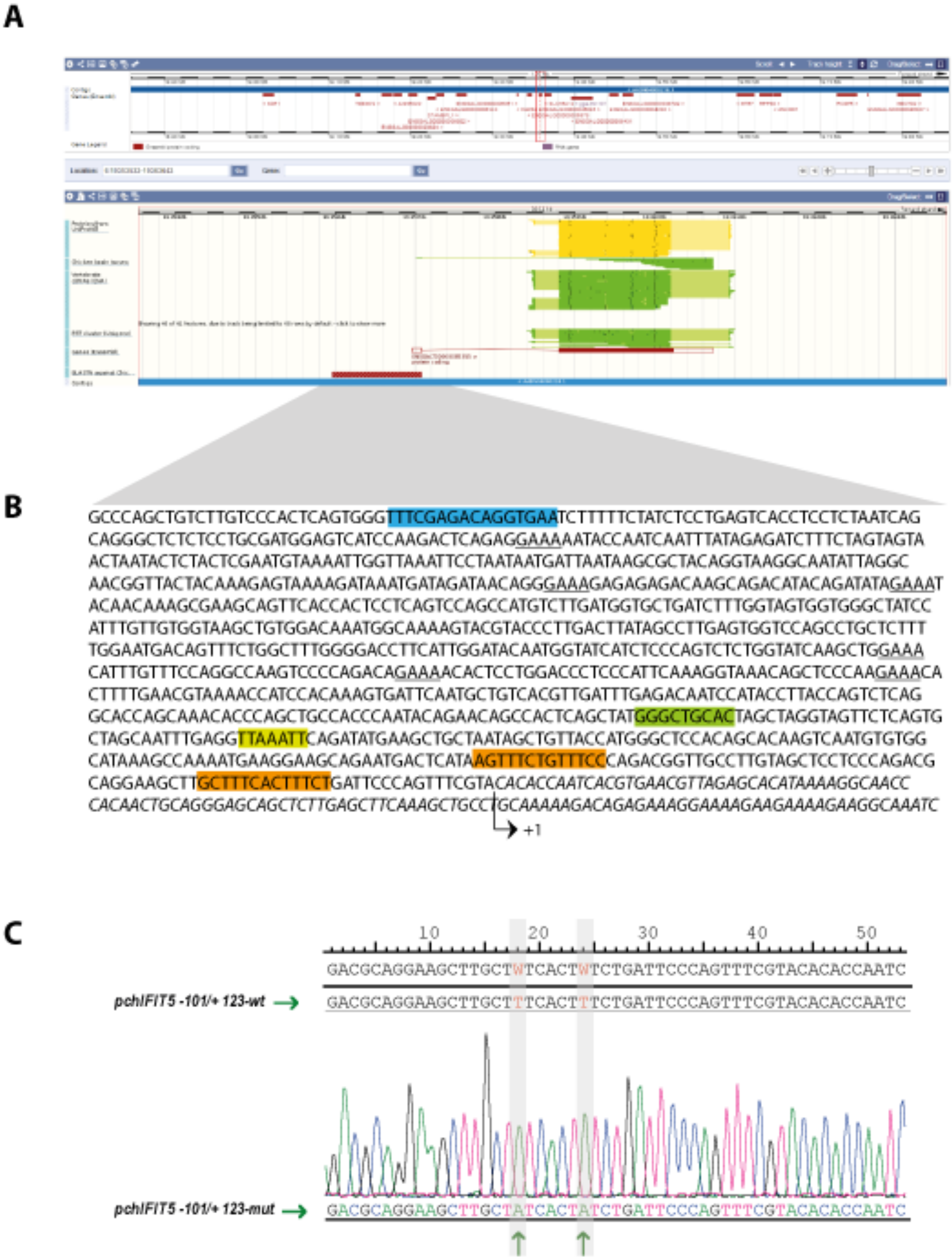

Figure S2 │ Promoter structures and putative *cis-acting* elements in the chIFIT5 gene. (**a**) An overall view of the gene structure of chIFIT5 in chicken genome. (**b**) A total of around 1.2 kb sequence upstream to the chIFIT5 gene was extracted and possible functional motifs are shown in the sequence. Two ISRE elements are marked orange, TATA box marked as yellow and SpeI site is marked with green background. GAS sequence is given blue colour whereas GAAANN sites are underlined. (**c**) Chromatograms of DNA sequencing showing mutated sites in the pchIFIT5-101/+123-wt and pchIFIT5-101/+123-mut constructs.

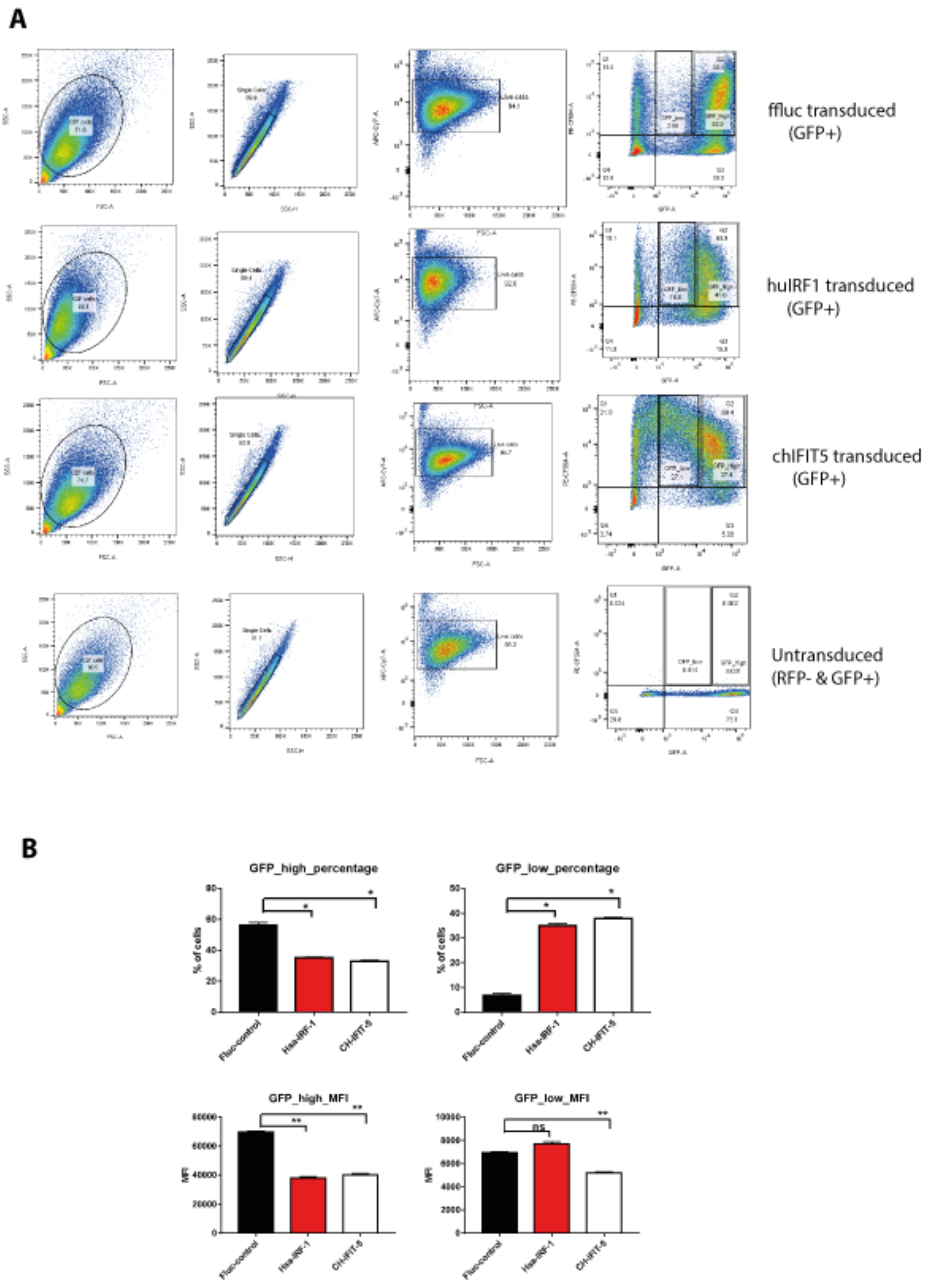

Figure S3│Gating strategy for marker (GFP+/RFP+) expressing cells. (**a**) FACS gating strategy for analysis of data shown in Figure 5 of main manuscript. (**b**) Percentage cell positivity and MFI in low and high GFP expressing cells.

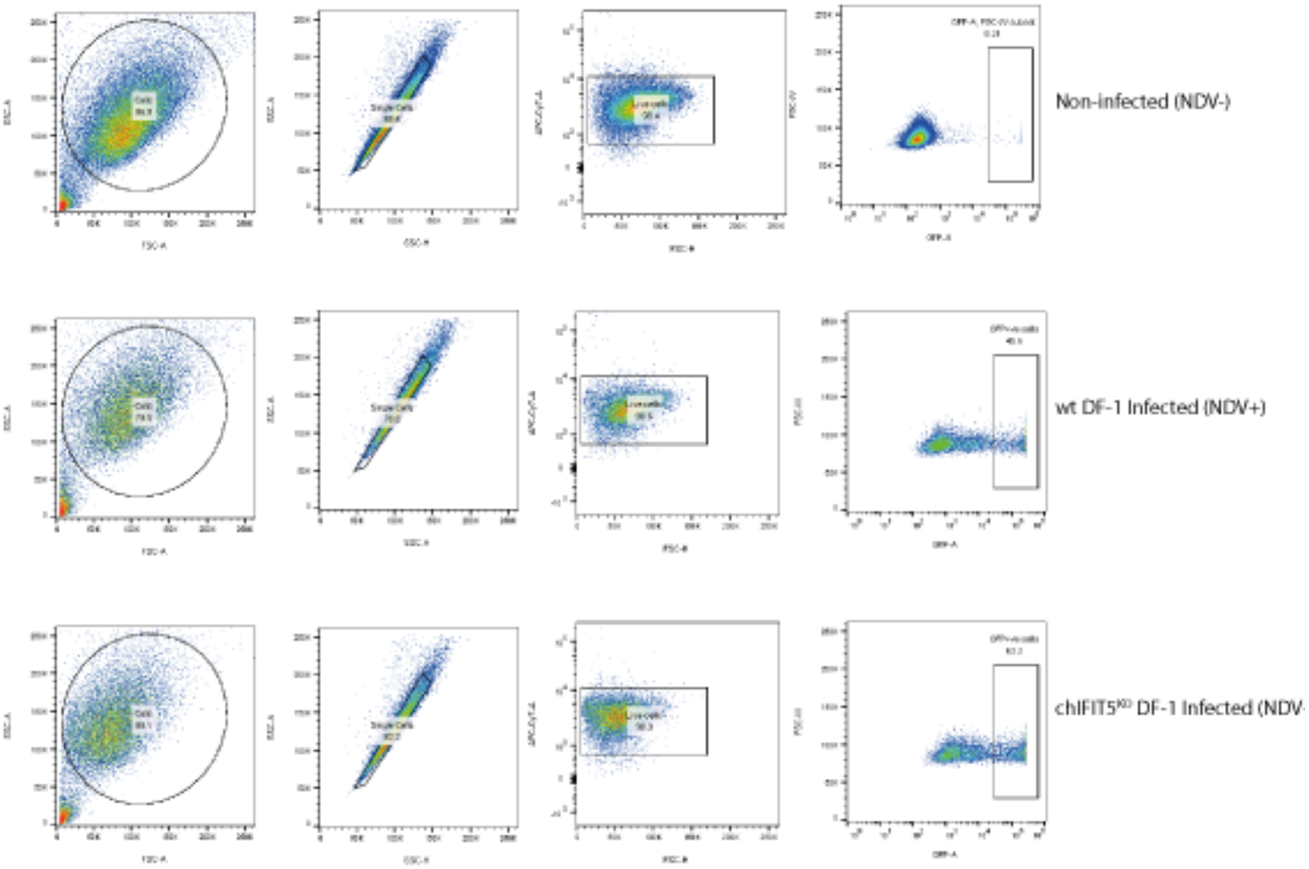

Figure S4│FACS gating strategy for analysis of data shown in Figure 6 of main manuscript for evaluation of antiviral effects in chIFIT5^{KO} DF-1 cells compared to wild type DF-1 cells.

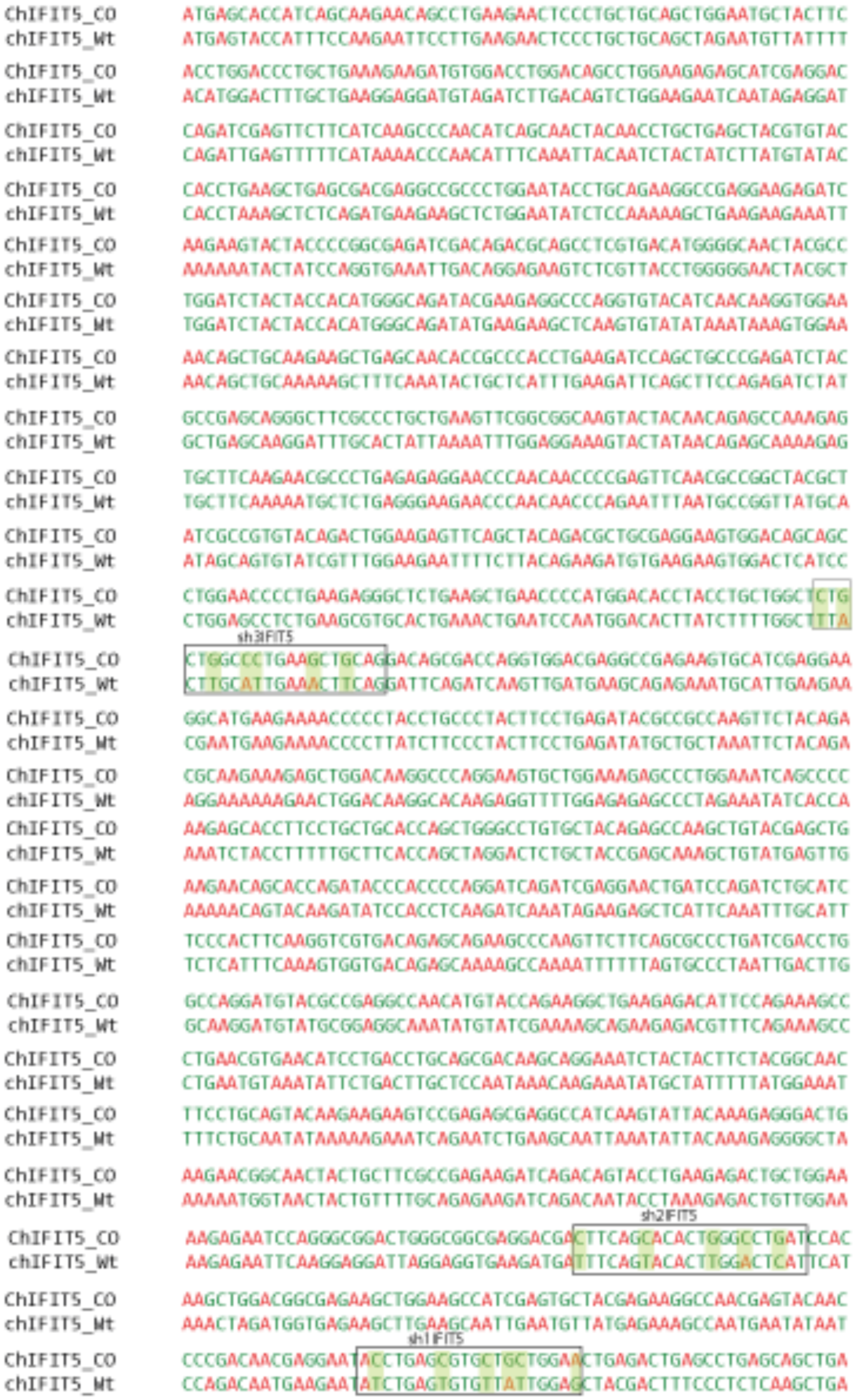

Figure S5│ Codon optimized-chIFIT5 used to generate shRNA-resistant IFIT5 expressing retroviruses. Positions of three shRNA targeting sites are highlights in wt and codon-optimized chIFIT5 genes.

Supplementary Table 1: Oligonucleotides used in this study.

Name of the primer	Sequence of the primer (5' to 3')	Purpose
28S-F	GGCGAAGCCAGAGGAAACT	qPCR
28S-R	GACGACCGATTTGCACGTC	-do-
Mx-F	CACTGCAACAAGCAAAGAAGGA	qPCR
Mx-R	TGATCAACCCCACAAGGAAAA	-do-
IFIT5-F	CAGAATTTAATGCCGGTTATGCAA	qPCR
IFIT5-R	TGCAAGTAAAGCCAAAAGATAAGTGT	-do-
chIFITlocus1-F	CCGACGGCGAACGCGCCCAGAA	Amplification of genomic gap
chIFITlocus1-R	GGGGTATTCGTGCAATTTCAGTCAA	-do-
chIFITlocus2-F	CATATTCGTCTCCGGGTCCACATT	-do-
chIFITlocus2-R	CTGCGGACGGGCTGACGGTCATCGG	-do-
chIFITlocus3-F	CGGCGCCAAGGCGGCTACGTAAGA	-do-
chIFITlocus3-R	TTTGTGCGAGGATAACGGTGCTGGC	-do-
chIFITlocus4-F	CGTACAGCAACACTACGGCAGACAGCAGGC CCCGCATCCTGCCGGGCTG	-do-
chIFITlocus4-R	TGCAAGCATGGCCACACGCCGCCTGACCAC AAGCACTGCTCTGGGACAT	-do-
chIFIT5-pLINK-F	ccGGATCCATGAGCACCATCAGCAAGAAC	Cloning of chIFIT5 in V5 tagged vector
chIFIT5-pLINK-R	gggGAATTCTCAGCTGCTCAGGCTCAGTC	-do-
chgIFIT5-F	GGAGGATGTAGATCTTGACAG	Amplification of genomic DNA for CRISPR/Cas9 InDel
chgIFIT5-R	CTTCCAAACGATACACTGC	-do-
pIFIT5-101mut-F	gaaactgggaatcagatagtgatagcaagcttcctgcgtct	Mutagenesis of promoter
pIFIT5-101mut-R	agacgcaggaagcttgctatcactatctgattcccagtttc	-do-
RCASA-IFIT5-F	CCCATCGATATGCTCGGAAAGCCGATCCCA AACC	Cloning of IFIT5 in RCASA(BP) vector
RCASA-IFIT5-R	CGCACGCGTTCAGCTGCTCAGGCTCAG	-do-
chIFIT5-sh1F	gagaggtgctgctgagcgcTATCTGAGTGTGTTATT GGAGtagtgaagccacagatgta	Cloning of shRNA for chIFIT5
chIFIT5-sh1R	attcaccaccactaggcaATATCTGAGTGTGTTATTG GAGtacatctgtggcttcact	-do-
chIFIT5-sh2F	gagaggtgctgctgagcgcTTTCAGTACACTTGGAC TCATtagtgaagccacagatgta	-do-
chIFIT5-sh2R	attcaccaccactaggcaATTTCAGTACACTTGGAC TCAT tacatctgtggcttcact	-do-
chIFIT5-sh3F	gagaggtgctgctgagcggTTACTTGCATTGAAACTT CAGtagtgaagccacagatgta	-do-
chIFIT5-sh3R	attcaccaccactaggcaTTTACTTGCATTGAAACTT CAG tacatctgtggcttcact	-do-
1hp-L	ggcggggctagctggagaagatgccttccggagaggtgctgct gagcg	Universal shRNA oligo
1hp-R	gggtggacgcgtaagaggggaagaaagcttctaaccccgctat tcaccaccactaggca	-do-
huIFIT5-pLINK-F	ccGGATCCATGAGTGAAATTCGTAAGGA	Cloning of human IFIT5 in expression vector
huIFIT5-pLINK-R	gggACTAGTTTAAATGGAAAGTCGGAGC	-do-
